# Shining a Light on Patterns of Photoperiod Sensitivity in Germination and Flowering Across Latitudes, Ecosystems and Functional Traits

**DOI:** 10.1002/ece3.71923

**Published:** 2025-08-27

**Authors:** Ashika Perrin, Angela T. Moles, Catherine A. Offord, Eve Slavich, Karen Zeng, Susan E. Everingham

**Affiliations:** ^1^ Evolution & Ecology Research Centre, School of Biological Earth and Environmental Sciences UNSW Sydney New South Wales Australia; ^2^ Australian PlantBank Botanic Gardens of Sydney New South Wales Australia; ^3^ Stats Central, Mark Wainwright Analytical Centre UNSW Sydney New South Wales Australia; ^4^ Institute of Plant Sciences University of Bern Bern Switzerland; ^5^ Oeschger Centre for Climate Change Research University of Bern Bern Switzerland

**Keywords:** germination, growth form, habitat, latitude, phenology, photoperiod sensitivity, phylogeny, seed flowering

## Abstract

In photoperiod sensitive plants, the timing of phenological events depends primarily on day length rather than temperature, precipitation or other environmental variables. This may make these photoperiod sensitive species less able to respond to climate change as their phenologies are more tightly controlled by day length conditions, which remain constant into the future, than by changing climatic conditions. We measured germination under three light treatments (short‐day, long‐day and equal light and dark) to quantify species' germination photoperiod sensitivity. We developed a novel metric that provides a continuous measure of germination photoperiod sensitivity for 67 plant species across a range of locations, habitats and growth forms. Of the 67 species, four species had significantly more seeds germinate in the long‐day treatment, three species had significantly higher germination in the short‐day treatment, and four species had significantly higher germination in the intermediate‐day treatment. We then used this metric to quantify the relationships between germination photoperiod sensitivity (as quantified from the laboratory tests and calculated metric) and phylogeny, seed size, growth form, latitude, leaf area index (LAI) and ecosystem type. We also quantified the relationships between flowering photoperiod sensitivity (as quantified by a literature review) and the same ecological/environmental predictor variables as were tested against germination photoperiod sensitivity. There were no significant relationships between photoperiod sensitivity in germination or flowering and species' biogeography, phylogeny or other functional traits. Our findings suggest that photoperiod sensitivity is likely to be important in a range of different locations and in different types of species.

## Graphical abstract

Our study investigated the role of photoperiod sensitivity in plant phenology, focusing on germination and flowering patterns across species in Australia and globally. We found that most species showed minimal photoperiod sensitivity in their germination, and there were no significant relationships between photoperiod sensitivity and factors like biogeography, phylogeny or other traits and environmental variables. Our findings suggest that predicting how photoperiod sensitivity will influence species' responses to climate change is challenging, highlighting the need for more empirical data.

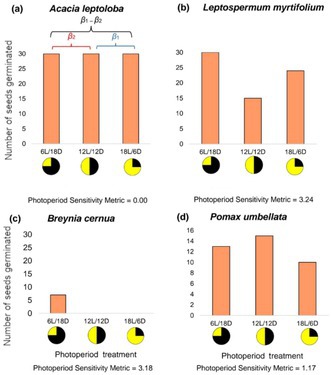

## 1 Introduction

The timing of phenological events such as budding, flowering and germination is crucial for successful plant reproduction (Basler and Körner 2012). Some plants have evolved a day length (photoperiod) sensitivity that cues or restricts phenological events (Jackson 2009). Along with other environmental cues such as temperature and precipitation changes, photoperiod sensitivity can prevent seeds from germinating (Gallagher 2013) or plants from flowering (Putterill et al. 2004) at the wrong time of year by inhibiting germination or flowering under certain day length conditions or initiating these processes when seasonal day length conditions are met. Photoperiod sensitivity may protect species from the adverse results of temperature‐related phenological shifts that are not always adaptive (Inouye 2008). However, warmer temperatures due to climate change are causing a shift in the timing of seasonal events, for example, shorter and milder winters and an earlier onset of spring (Cooper 2014; Ernakovich et al. 2014; Stine et al. 2009), and highly photoperiod sensitive species (species that are promoted or delayed in their phenology by specific durations of light and darkness) may not respond in altered germination or flowering timing to changing environmental conditions (Zeng et al. 2024). This may lead to species delaying germination or flowering to match particular day length conditions and failing to germinate or flower with earlier spring conditions. Species that match their phenologies more tightly to day length conditions (photoperiod sensitive species) than other environmental cues may not optimise their germination or flowering in earlier springs or summers. As a consequence, under future climate change, photoperiod sensitive species may be outcompeted by other native or introduced plants that are not constrained by day length (Saikkonen et al. 2012).

Photoperiod sensitivity in flowering time has been quantified for a large number of species across the globe (Zeng et al. 2024). Flowering photoperiod sensitivity patterns may not be directly transferable to those of photoperiod sensitivity in germination, for which we have limited data. Photoperiod sensitivity in germination has been quantified for only a small number of species in the northern hemisphere (Imaizumi et al. 2017; Kauth et al. 2008; Lin and Wang 2017), as well as agricultural species (Hu et al. 2024), and it has been largely unexplored in the southern hemisphere (but see Paton 1978; Welgama et al. 2019). Studies have also shown that germination in some species may not be responsive to photoperiod (Stubbendieck and McCully 1976; Imaizumi et al. 2017; Wagner and Oplinger 2017). Southern hemisphere plants may respond differently in their germination or flowering time to photoperiod than northern hemisphere plants because of the lack of extreme low temperatures across most of the southern hemisphere (due to lower latitudes of many of the continents and the higher ratio of ocean to land mass in the southern hemisphere). By gaining fundamental knowledge about germination and flowering photoperiod sensitivity in understudied southern hemisphere species, we can contribute to conservation efforts. For example, fundamental photoperiod sensitivity germination data may be used by seed scientists to maximise the germination of seeds that may be used in *ex situ* seed banking or for propagation and translocation of species for regeneration efforts.

We experimentally quantified germination photoperiod sensitivity for a range of Australian species and collated flowering photoperiod sensitivity using a literature review for additional species across the globe. We then used our photoperiod sensitivity data to test what types of habitats, environmental conditions and species characteristics are associated with strong photoperiod requirements in both germination and flowering. If we can predict photoperiod sensitivity using easily measured factors such as phylogeny, seed mass or growth form, this information can be applied to conservation efforts in determining species' preferred photoperiodic germination conditions. Alongside data on other environmental cues and inhibitors of germination and flowering time, such as temperature and precipitation, resolving species photoperiod sensitivity will allow researchers to have a greater understanding of species that are vulnerable to climate change in the future.

Certain taxa, such as Euphorbiaceae and some species of Brassicaceae, have been found to have photoperiod‐sensitive flowering time (Adeyemo et al. 2019; Golembeski et al. 2014; Hu et al. 2016; Leijten et al. 2018). However, Australia's flora includes many families for which germination photoperiod sensitivity has never been quantified, such as Proteaceae and Ericaceae. Our study aimed to determine whether there were any phylogenetic patterns in photoperiod sensitivity in germination across taxa. It is vital to determine whether there are any phylogenetic trends in germination photoperiod sensitivity, as this will impact other trait and habitat relationships. It will also indicate whether a species' germination photoperiod sensitivity can be predicted from taxonomy.

While studies have shown that sensitivity to light quantity and quality increases as seed size decreases (Aud and Ferraz 2012; Grime et al. 1981; Milberg et al. 2000; Wu et al. 2013), it is not clear whether a similar pattern in germination occurs in response to photoperiod. We tested the hypothesis that small‐seeded species tend to be more photoperiod sensitive in their germination than larger‐seeded species. Existing studies on this topic have only examined a small number of species and show contradictory results. Some studies have found larger seeds to be more independent of photoperiod in their growth and germination than smaller seeds (Mandal et al. 2008; White et al. 1992), but some extremely small seeds for particular species were completely unaffected by photoperiod (Stubbendieck and McCully 1976).

We also tested the hypothesis that trees have lower photoperiod sensitivity in germination than do herbs and shrubs. This prediction is based on the fact that trees tend to have longer reproductive lifespans and thus may be less sensitive to the failure of a given year's seed output. Consistent with this idea, Zeng et al. (2024) found that annual and herbaceous species were more likely than perennial or woody species to have photoperiod‐sensitive flowering. However, there has been no previous cross‐species test of this idea for photoperiod‐sensitive germination.

Next, we tested a series of hypotheses about biogeographic patterns in photoperiod sensitivity. We aimed to determine if there were any relationships between germination photoperiod sensitivity, the latitude or habitat openness of a species' native habitat, or whether a species was from an alpine versus non‐alpine ecosystem. This information may help to better identify which ecosystem types are more likely to have species whose photoperiod requirements prevent them from shifting their phenology in response to climate change.

Although it has not been tested across a broad geographic range or numerous species, we predicted that species at higher latitudes (further from the equator) would tend to be more photoperiod sensitive in their germination than species at lower latitudes. Stronger seasonal changes in day length occur at higher latitudes (Saikkonen et al. 2012). Higher latitudes also have more extreme low temperatures that might impose a strong selective pressure on species to have photoperiod sensitive germination. Previous studies of flowering time have shown a higher photoperiod sensitivity at higher latitudes; however, these studies only incorporate a small number of species or across a narrow latitudinal range (Amasino 2010; Samach and Coupland 2000) and may not directly translate to patterns in germination photoperiod sensitivity. Latitudinal trends in germination photoperiod sensitivity have been investigated in some species; however, most of these studies used seeds from a small latitudinal range and have shown varied results. Some single‐species studies have found greater photoperiod sensitivity at higher latitudes (Bevington 1986; Upadhyaya et al. 2012). However, as low temperatures become milder at lower latitudes, temperate species that cue on temperature throughout most of their range may shift to insolation‐driven phenology (Borchert et al. 2005) and photoperiod has been shown to be an important driver of flowering time in tropical species (Rivera and Borchert 2001). Some studies have shown no relationship between latitude and photoperiod sensitivity (Paton 1978; Welgama et al. 2019). Snowmelt, phylogeny and other functional traits may confound latitudinal patterns in germination photoperiod sensitivity as they buffer both daily light and temperature fluctuations in species in alpine environments and they can control phenological responses at high latitudes (Inouye 2008; CaraDonna et al., 2014).

Across ecosystems, plants' exposure to light varies substantially due to differences in canopy structure (Depauw et al., 2020), which may influence how tightly bound to photoperiod a species is in its germination. We hypothesised that photoperiod sensitivity in germination would decrease with increasing canopy cover, since species growing under a dense canopy are exposed to lower light conditions and thus weaker photoperiodic cues for germination. Several studies have investigated the effects of light quality on germination in open and/or closed environments (Bell et al. 1999; Maarel & van der Maarel and Franklin 2012; Pezzani and Montana 2006; Vàzquez‐Yanes and Orozco‐Segovia 1990); however, there is very limited research on the effects of photoperiod in ecosystems with varying degrees of canopy cover.

Alpine ecosystems experience low temperatures and harsh seasonal winters (Billings 1974), placing greater selective pressure on species' germination at the right time of year. Species may therefore be more photoperiod sensitive and require a longer day length to germinate. This has been observed in Alaskan alpine plants, where simulated short‐day autumn conditions significantly inhibited germination, whereas spring long‐day conditions encouraged germination (Densmore 1997). There have also been studies that show the tight linkage of some species' flowering and fruiting with photoperiod in alpine zones of the northern hemisphere (Keller and Körner 2003) and the southern hemisphere (Venn and Morgan 2007). However, there is limited data and evidence on these patterns across species within alpine regions. Therefore, we hypothesised that alpine species would tend to be more photoperiod sensitive in their germination than non‐alpine species. Alpine species are thought to face greater risks under climate change (Dullinger et al. 2012) and if alpine species' germination is tightly correlated with photoperiod, many species could be vulnerable to extinction under unfavourable climatic conditions.

Although flowering phenology is generally better studied than germination phenology (e.g., Everingham et al. 2023), we have surprisingly little information about broad‐scale trends in flowering photoperiodism. The only analysis of which we are aware found stronger photoperiod sensitivity in herbaceous and annual species than in woody or perennial species, and a phylogenetic signal in photoperiod sensitivity (Zeng et al. 2024). Thus, our final goal was to test whether photoperiod sensitivity in flowering is related to three key variables that have yet to be tested, including seed mass, latitude or leaf area index. Similar to our hypothesised patterns in germination photoperiod sensitivity, these three variables may pose various environmental pressures and selection on species to be strongly photoperiod sensitive or not.

In this study, we quantify germination photoperiod sensitivity in 67 Australian species from 26 families across a broad range of seed masses (< 0.001–3.08 g), growth forms (herbs, shrubs, climbers and trees), latitudes (16° S—36° S), and both alpine and non‐alpine habitats. In addition to providing basic information about the prevalence and direction of photoperiod sensitive germination in southern hemisphere plants, we asked whether there were broad patterns in the types of species that may be more photoperiod sensitive in their germination than others, including testing if different families or genera are more photoperiod sensitive than others, or if species with particular traits and growth forms have higher photoperiod sensitive germination. We then aimed to determine if species from particular ecosystems (closed canopy systems or alpine ecosystems) have higher germination photoperiod sensitivity. Finally, we aimed to determine if these ecological and environmental patterns in germination photoperiod were mirrored in relationships between photoperiod sensitivity in flowering in a global flowering dataset and the same abiotic and biotic factors.

## 2 Materials and Methods

### 2.1 Seed Collection

**FIGURE 1:**
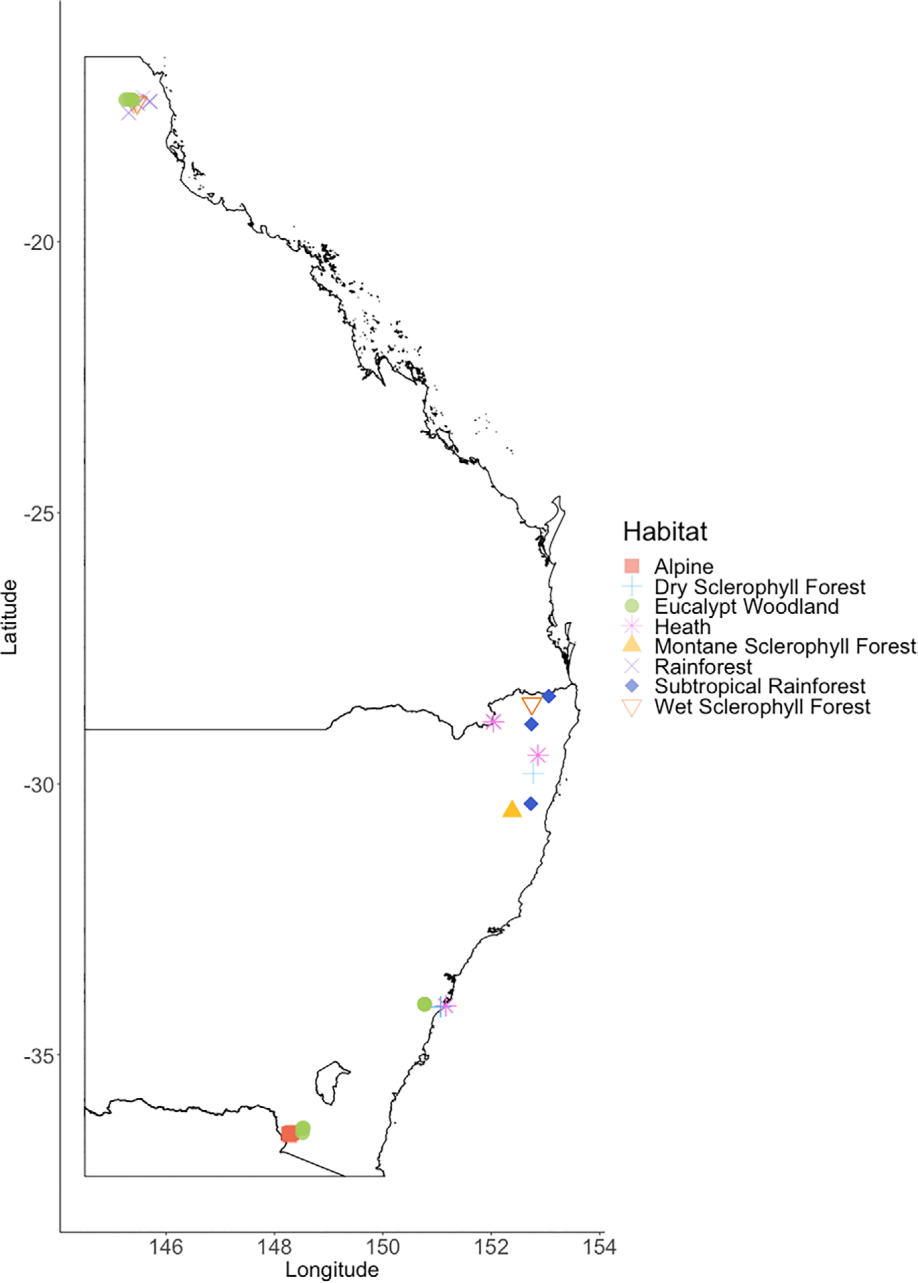
[Size = single column] Field sites across eastern Australia where seed collection from native plant species occurred. Map created in R Studio (version 3.6.0) using ‘ggplot2’ (Wickham, 2016) and open‐source shape file of Australia from Geoscience Australia (https://data.gov.au/dataset/ds‐ga‐a05f7892‐eae3‐7506‐e044‐00144fdd4fa6/details?q=, retrieved 17 May, 2020).

We collected fresh seeds from 98 native species from eastern Australia (Figure 1, see Appendix S1, Table S1.1 for a full species list). Study sites ranged from 16° S in far north Queensland to 36° S in southern New South Wales (spanning approximately 2200 km in latitude) and spanned multiple ecosystems including heathland, woodland, rainforest, and alpine herb‐fields (Figure 1, Appendix S1). A handheld GPS (Garmin Oregon 600 t) was used to record the longitude (°E), latitude (°S), and elevation (m) of each site.

Seed collection followed the Cornelissen et al. (2003) protocol. That is, seeds were collected from five or more individual plants for each species. Where possible, seeds were evenly collected across individuals and within individuals (i.e., from numerous branches) to ensure samples were reflective of true wild‐type characteristics (Meyer and Monsen 1993). Depending on seed availability, for each species, anywhere from 16 to over 500 seeds were collected (see Appendix S1 for total seeds used per species).

### 2.2 Seed Processing, Weighing and Germination Testing

We cleaned the collected seeds from their respective fruits/cones/pods, and the woody endocarps of some species were cracked to remove the seeds from the fruits. Cones of *Banksia* species were heated in an oven (Labec Laboratory Equipment PTY LTD) at 120°C for half an hour to release the seeds (Harvey 1995). We also scarified seed coats of Fabaceae species from the genera: *Acacia*, *Austrosteenisia* and *Vachellia*, before germination (Pound et al. 2015). We weighed 50 seeds from each species to determine average fresh seed mass (mg) using a micro‐balance (Mettler Toledo PTY LTD, accuracy: 1 × 10^−4^ g).

We quantified photoperiod sensitivity using an experimental day length manipulation method. Three photoperiod treatments were used, including: 6 h of light, 18 h of dark (6 L/18D; short‐day); 18 h of light, 6 h of dark (18 L/6D; long‐day); and 12 h of light, 12 h of dark (12 L/12D; equal light, equal dark). These conditions were maintained in three incubators (Labec Laboratory Equipment PTY LTD), each of which was illuminated with cool white, fluorescent tubes and kept at a constant temperature of 20°C, and the photon flux density was constant (between 6 and 15 μmol m^2^ s^−1^; measured with a light metre; LI‐COR 250A) between incubators. Incubators remained sealed at all times, and sampling was done within the illuminated hours, preventing any uncontrolled light interference during dark periods.

A total of 6–50 seeds (see Table S1.1 for sample sizes) of each species were placed in Petri dishes containing an agar solution (0.8% w/v water agar) in the three different incubators with the photoperiod treatments. The number and density of seeds within each Petri dish were equal across the three photoperiod treatments within each species and were determined for each species based on seed size (i.e., smaller seeds were placed in larger numbers on Petri dishes and vice versa).

Seeds remained in the incubators until all had germinated, or until 90 days had elapsed. We scored germination every 2 days for 90 days (March 2020–June 2020). Seeds were counted as germinated upon the emergence of a radicle (International Seed Testing Association 2010). Any non‐germinated seeds were tested for viability at the end of the 90‐day period. We tested viability using a microscopic cut test analysis and staining with 1% 2,3,5‐triphenyltetrazolium chloride following the Millennium Seed Bank (MSB) procedure, which stains the embryo red if respiring and is a transferable and reliable method across all plant species (Egido et al. 2017).

### 2.3 Literature Review for Global Flowering Photoperiod Sensitivity

To determine if global flowering photoperiod sensitivity was also related to latitude, seed mass, leaf area index and environment type, we compiled flowering photoperiod sensitivity data from the literature. We searched ISI Web of Science using the keywords ‘flower*’ in combination with the words ‘photoperiod*’ OR ‘long day’ OR ‘short day’ OR ‘day neutral’ in all document types and languages from 1900 to 2020, which returned 2782 papers. Additional papers and reference books were added from references in literature reviews on photoperiod sensitivity. We manually read and recorded the photoperiod sensitivity status of each species tested within each paper, excluding populations selectively bred for agricultural purposes and plants without a yearly flowering cycle. We recorded each species' photoperiod sensitivity based on four categories (long day, short day, other photoperiod and no photoperiodism). We found 832 records of categorised photoperiod sensitivity covering 747 species, which we attempted to determine geographical coordinates of based on information provided in the source publication. We were however unable to identify seed source in many publications, leaving us with 135 records representing 70 species consisting of 45 long day, 50 short day, 32 day neutral and 8 other records (Appendix S2, Table S2.1). We collected seed mass data for each species using the Kew Seed Information Database (Royal Botanic Gardens Kew, 2021).

### 2.4 Environmental Variables

Canopy cover for each species for the germination photoperiod sensitivity analysis and flowering photoperiod sensitivity analysis was determined using a continuous measurement of leaf area index, the total one‐sided area of photosynthetic tissue per unit ground surface (Sabol et al. 2014). Leaf area index data were downloaded from Copernicus Global Land Service (version 2.2.1) and synthesised in QGIS (version 3.14.15 ‘Pi’) to retrieve LAI values for each species' sampling location (Tables S1.1 and S2.2), at a spatial resolution of 300 m.

### 2.5 Data Analyses

All statistical analyses were performed in R (version 4.0.0) in R Studio statistical software (R Core Team 2020). All code and data are freely available at https://anonymous.4open.science/r/Trends‐in‐Germination‐Photoperiod‐326E/.

Germination photoperiod sensitivities for each of the 98 species collected in our fieldwork were calculated as the standard deviation of the estimated treatment means (Equation 1) from Bayesian Generalised Linear Models (Bayesian GLM), using the function ‘bayesglm’ in the ‘arm’ package (Gelman et al. 2020). We took the Bayesian GLM approach to constrain estimated effect sizes that tend to occur in ‘zero’ cases—that is, where there was no germination occurring in any of the 3 day length light‐treatments in a particular species. Where there was not zero seed germination in any treatment‐species combination, these models produce very similar coefficient estimates to a frequentist GLM. These models were run with each species' binary (germinated/non‐germinated) germination outcome per seed as the response variable and photoperiod treatment (long‐day, short‐day, equal‐day) as a categorical predictor variable. Across all species, we used Bayesian parameterised GLMs with a prior scale of 2.5 (as suggested by Gelman et al. 2008), as this regularised extremely high parameter estimates that occurred when there was zero germination in some treatment levels. The effect of this prior scale was that the absolute values of coefficients were extremely unlikely to be estimated above five; we did not believe such changes were likely.

$$\text{Photoperiod Sensitivity Metric}=\sqrt{\left(\frac{{\left(\beta_{1}\right)}^{2}+{\left(\beta_{2}\right)}^{2}+{\left(\beta_{1}-\beta_{2}\right)}^{2}}{3}\right)}$$

Equation 1 Photoperiod sensitivity metric calculated from the model parameters as the standard deviation of the estimated treatment means from the Bayesian Generalised Linear Models. In this equation: *β*_1_ represents the difference between the probability of germinating under the 18 L/6D treatment and the 12 L/12D treatment; *β*_2_ represents the difference between the probability of germinating under the 6 L/18D treatment and the 12 L/12D treatment; and *β*_1_—*β*_2_ represents the difference between the probability of germinating under the 18 L/6D treatment and the 6 L/18D treatment. The differences in probabilities of germination in each treatment were divided by 3 due to the fact that there were three light treatments.

**FIGURE 2:**
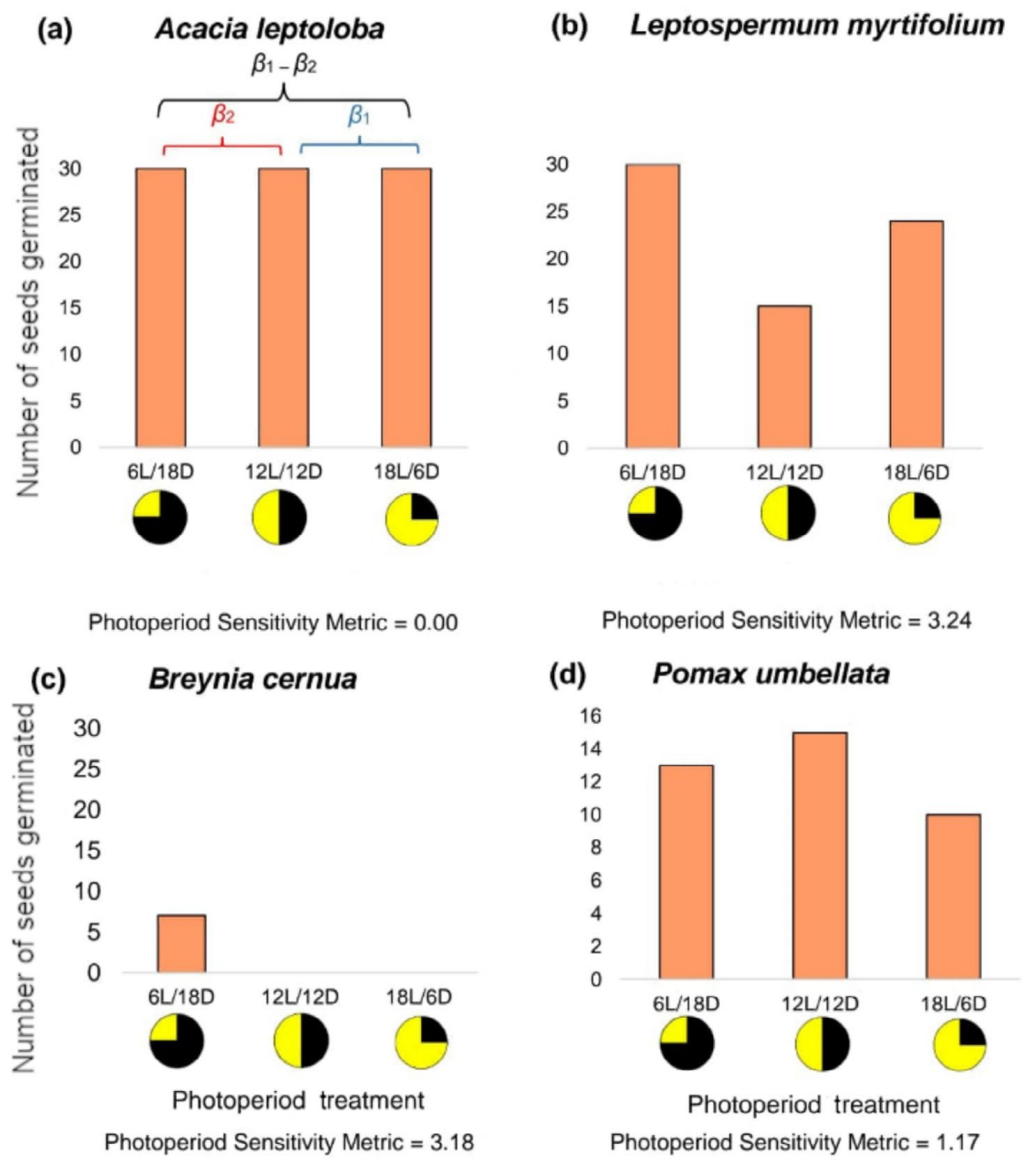
[Size = single column] Examples of four species with widely varying germination data to provide examples of different species' results of our Photoperiod Sensitivity Metric (PSM) calculation. Our PSM was calculated from the standard deviation of the estimated treatment means from the Bayesian Generalised Linear Models (see Equation 1). The x axes indicate the various photoperiodic treatments that were applied, where the number and ‘L’ corresponds with the hours of light received, and the number and ‘D’ corresponds with the hours of darkness received. The pie charts that are directly underneath visually articulate the applied treatments, showing the ratio of light (in yellow) to darkness (in black). The maximum number on each y axis is respective to the maximum number of seeds that were placed in all three photoperiodic treatments for that species. For example, (a) *A. leptoloba*, (b) *L. myrtifolium* and (c) *Breynia cernua* each had 30 seeds placed in each treatment and (d) *Pomax umbellata* had 16 seeds placed in each treatment.

Using the photoperiod sensitivity metric, a species with a large difference in germination success among the three treatments, or between one treatment and the equal‐day treatment (12 L/12D), would have a higher photoperiod sensitivity metric (Figure 2). In contrast, a species with equal germination across all three treatments would have a photoperiod sensitivity metric closer to zero (Figure 2). All results of species photoperiod sensitivity metrics are available in Appendix S3, Table S3.1.

We extracted the standard error from the Bayesian GLM models for each species to use for weighting each species' photoperiod sensitivity metric in further analyses, as each species had differing sample sizes and variance. The standard error was calculated as the standard deviation of 10,000 iterations of the sensitivity metric simulated in a parametric bootstrap using the ‘rmvnorm’ function in the ‘mvtnorm’ package (Genz et al. 2020).

Thirty‐one of the 98 species did not have sufficiently high germination across all treatments to accurately quantify photoperiod sensitivity (< 5% germination in all treatments, or less than five seeds germinated across all three photoperiod treatments). This may be due to other constraining factors on their germination in vitro. These species' germination data are reported in Appendix S3. However, they were excluded from further analyses, resulting in our proceeding analyses being performed on 67 species (from 50 genera, across 29 families).

To test the hypothesis that photoperiod sensitivity in germination is phylogenetically conserved, we used a phylogenetic generalised least squares (PGLS) framework to estimate the covariance of photoperiod sensitivity across our 67 species. This covariance matrix was obtained from the relationships and branch lengths of the species after being pruned from the mega‐tree created by (Smith and Brown 2018) using the ‘phylo.maker’ function in the package ‘V.PhyloMaker’ (Jin and Qian 2019). The covariance matrix and branch lengths were then used to test for a phylogenetic signal in photoperiod sensitivity across our species using Pagel's *λ* and Blomberg's *K* (Münkemüller et al. 2012). One species (*Hakea dactyloides*) had seeds collected from two sites with differing photoperiod sensitivity metrics for each site. For this species, in the phylogenetic analysis we used the average photoperiod sensitivity metric from the two sites.

To test the hypotheses about relationships between photoperiod sensitivity and potential geographic and ecological predictors, we performed separate Generalised Linear Models (GLM) with the ‘glm’ function and the package ‘lme4’ (Bates et al. 2015). We used the absolute value of our pre‐calculated photoperiod sensitivity metric (from Equation 1) as the response variable, and seed mass (log_10_ transformed), growth form (determined for each species from the NSW Plant Information Network System; Royal Botanic Gardens and Domain Trust, 2020 and removing ‘climber’ as a growth form as only two species had this growth form), latitude, leaf area index and alpine versus non‐alpine habitat as the separate predictor variables in each respective model. The GLMs including all species were Gamma distributed and weighted by the variance of the photoperiod sensitivity metric (calculated as 1/(standard error of the Bayesian GLMs)^2^).

We used a similar approach for the flowering time photoperiod sensitivity as the germination photoperiod sensitivity Generalised Linear Models previously. We replaced the continuous photoperiod sensitivity metric for a categorical response variable, using extracted published species classifications as either photoperiod sensitive or insensitive in their flowering.

### 2.6 Methodological Considerations of Using Outputs From Bayesian Models in Frequentist Models

We used the outputs from Bayesian models and calculated a species' photoperiod sensitivity metric as the response variable in frequentist models in a two‐step process. Separating the method into two steps (i.e., calculating species‐wise photoperiod sensitivity metrics first, before looking at trends in photoperiod sensitivity with environmental variables) provides future researchers with a simple analytical tool in the first step (the Bayesian analysis with photoperiod sensitivity metric calculation) to calculate a continuous metric of photoperiod sensitivity. We argue it is easier in this framework to visualise and communicate the relationships between metrics and our variables of interest. We used the standard error of the species‐wise photoperiod sensitivity metric calculated in the first step as weights in the second stepto propagate the uncertainty through to the second model. As a supplementary analysis, we ran Bayesian Models using an ‘interaction‐GLMM’ approach, with germination as the response variable (binomial), and each environmental/ecological variable as predictor variables interacting with the photoperiod treatment. We used the ‘glmmTMB’ package (Brooks et al. 2017) in R to run these models. We also included a random intercept for species and a random slope for treatment per species. From the regression coefficients of this model, we were able to construct the equivalent photoperiod sensitivity metrics and slope of the photoperiod sensitivity metric with the same selected environmental/ecological variables and parametrically bootstrap the standard error/confidence interval of this slope. We found our results were not sensitive to the methodology employed (Appendix S4).

## 3 Results

**FIGURE 3:**
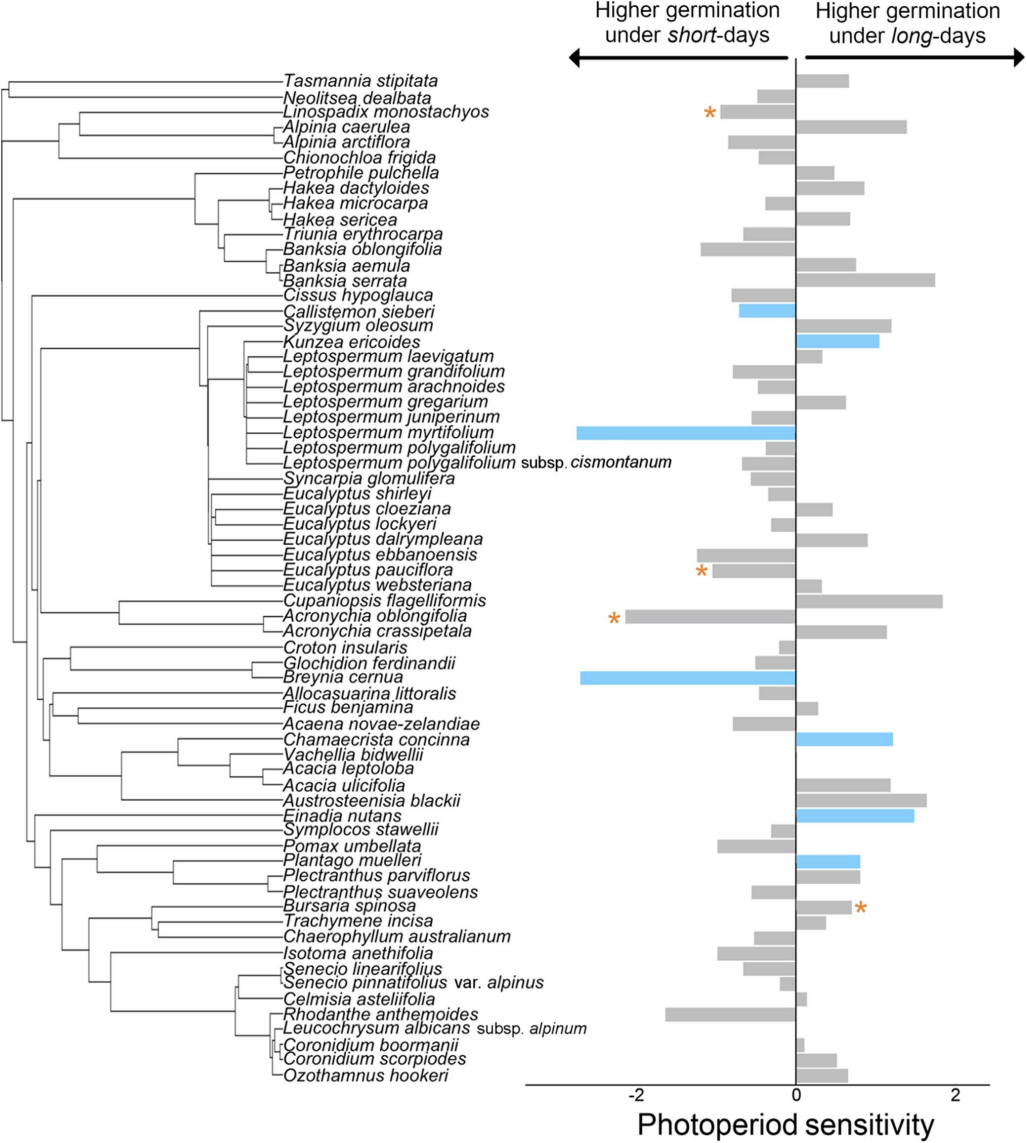
[Size = double column] The photoperiod sensitivity of 67 native species from eastern Australia (*Hakea dactyloides* had two measurements of photoperiod sensitivities from different localities and they are averaged here for phylogenetic analysis). The length of the bars represents the photoperiod sensitivity metric, which is calculated as the standard deviation of the germination under long‐days, short‐days and equal‐days (see methods for details of classification). Species with higher germination under long days are indicated by positive photoperiod sensitivity values while species with higher germination under short days are indicated with negative photoperiod sensitivity values. Species with significant photoperiod sensitivity are indicated by the light blue colour and species with non‐significant photoperiod sensitivity are indicated by the colour grey. Note that there were four species with significantly higher germination under intermediate day conditions (indicated by an orange asterisk) and these were then designated either short‐ or long‐day in sign (negative or positive) based on what germination was higher out of these two categories. Where species' photoperiod sensitivities are blank, this indicates equal germination across all three treatments, hence a photoperiod sensitivity index close to zero.

Only eleven of the 67 species showed significant photoperiod sensitivity in their germination (Figure 3). Of these, three species (*Breynia cernua*, Phyllanthaceae; *Callistemon sieberi*, Myrtaceae; and *Leptospermum myrtifolium*, Myrtaceae) had higher germination under short days than under long days. Four species (*Chamaecrista concinna*, Fabaceae; *Einadia nutans*, Amaranthaceae; *Kunzea ericoides*, Myrtaceae; *Plantago muelleri*, Plantaginaceae) had higher germination under long days than under short days. Four species had higher germination under intermediate day‐lengths (*Acronychia oblongifolia*, Rutaceae; *Bursaria spinosa*, Pittosporaceae; *Eucalyptus pauciflora* , Myrtaceae; and *Linospadix monostachyos* , Arecaceae). There were also three species that had equal germination across all three treatments, giving them a photoperiod sensitivity metric of 0 (*Acacia leptoloba*; Fabaceae, *Leucochrysum albicans* subsp. *Alpinum*, Asteraceae; and *Vachellia bidwillii*, Fabaceae; Figure 3).

No significant phylogenetic signal in photoperiod sensitivity was found (Pagel's *λ* = 0.000067, *p* = 1.00; Blomberg's *K* = 0.119, *p* = 0.53; Figure 3). That is, closely related taxa were not more likely to have similar photoperiod sensitivity.

**FIGURE 4:**
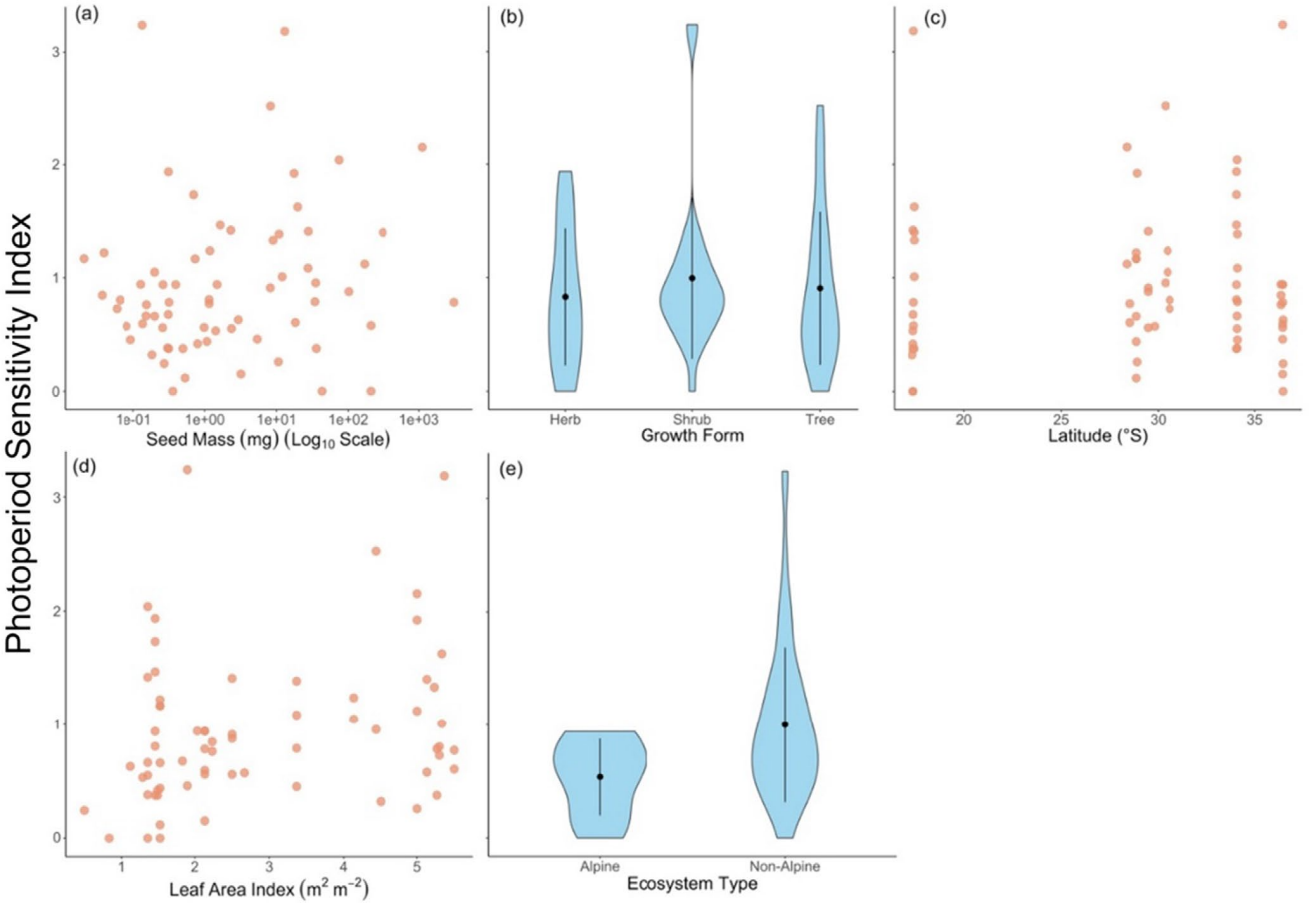
[Size = double column] No significant relationships were found between photoperiod sensitivity (calculated as an index calculated from the standard deviation of the estimated treatment means from Bayesian Generalised Linear Models) and seed mass, growth form, latitude, leaf area index or ecosystem type (alpine vs. non‐alpine). (a), (c) and (d) show scatterplots, and (b), (e) are violin plots showing the distribution of data with the mean and standard deviation expressed within the plots.

Counter to our hypotheses, we found no significant relationships between germination photoperiod sensitivity and seed size (*R* ^2^ = 0.01; *p* = 0.60; Figure 4a), growth form (*R* ^2^ = 0.10; *p* = 0.62, Figure 4b), latitude (*R* ^2^ = 0.001; *p* = 0.84; Figure 4c), canopy cover (LAI; *R* ^2^ = 0.05; *p* = 0.20; Figure 4d) or whether species were alpine or non‐alpine (*R* ^2^ = 0.06; *p* = 0.13; Figure 4e).

The lack of significant associations between germination photoperiod and putative predictor variables was also seen for flowering photoperiod sensitivity. Flowering photoperiod sensitivity was not significantly related to latitude (*R* ^2^ = 0.03, *p* = 0.14), leaf area index (*R* ^2^ = 0.12, *p* = 0.45) or seed mass (*R* ^2^ = 0.15, *p* = 0.60).

## 4 Discussion

Photoperiod sensitivity in germination and flowering is not significantly related to biogeography, phylogeny or functional traits. That is, we still do not have a way to predict which species are most likely to have photoperiod sensitive germination or flowering time. This is important, as photoperiod sensitive species may be limited in their ability to change their germination or flowering time in response to changing conditions (Ernakovich et al. 2014; Pagter and Arora 2013; Rawal et al. 2015; Zeng et al. 2024), and might thus be more at risk under a changing climate. A shortcut to predicting vulnerable species would have been useful, but for now, there is no way around the requirement for new empirical data on photoperiod sensitivity.

Only 12 out of the 67 species of Australian plants included in our study showed significant photoperiod sensitivity in their germination (Figure 3; Appendix S3). In contrast, studies in the northern hemisphere have shown substantially higher proportions of species that have strong effects of day length on their germination (Fosket and Briggs 1970; Lin and Wang 2017; Stearns and Olson 1958; Vaartaja 1956). Low levels of photoperiod sensitivity may prevent seedling failure under changed conditions and prevent species from being outcompeted by other native or introduced species (Saikkonen et al. 2012). The low level of photoperiod sensitivity in Australia is encouraging as it suggests that Australian plants may be able to change their germination timing in response to climate change, as they are not constrained to only germinate under particular day‐length conditions. However, plastic phenological responses to temperature and other environmental stimuli may not always be adaptive, so a lack of photoperiod sensitivity could also lead to negative outcomes for species' reproduction and survival. The photoperiods used in our experiments are more pronounced than natural conditions as we manipulated day lengths of 6 h (short‐day) and 18 h (long‐day) in order to quantify any photoperiod response under controlled conditions. Variation in day length between the longest and shortest day length in Australia is lower, with around two to five hours difference between the minimum and maximum day lengths. Therefore, we might expect that the effects of photoperiod in natural conditions are even less pronounced. The low photoperiod sensitivity in Australian species might reflect the importance of germination stimuli that are not associated with day length, such as large rainfall events in drylands or fire. It may also reflect the highly plastic nature of germination within species that our controlled, in vitro experiment does not capture (Zhang et al. 2022) and future research into the plastic nature of germination photoperiod sensitivity is needed to resolve these effects.

Another possibility is that the low prevalence of photoperiod sensitivity in germination in Australian plants is related to the relatively mild minimum temperatures in Australia (Alexander and Arblaster 2017). If a seed germinates at any given time of the year in many regions, seedlings have a low chance of damage from frost. This could lead to associated weaker selective pressure against seeds that germinate at the wrong time of year. If this is the case, we might expect other parts of the world (particularly the southern hemisphere and the tropics, where minimum temperatures tend not to be very low; Richter 2016) to also have very low rates of photoperiod‐sensitive germination. However, other research has shown that species occurring in regions with milder minimum temperatures switch to or rely on photoperiod‐cued phenological events such as flowering (Borchert et al. 2005). Gathering data for species from tropical systems and from other southern hemisphere regions such as South America, New Zealand and Southern Africa would be a worthwhile direction for future research and to disentangle this relationship for germination.

Our study was the first to quantify photoperiod sensitivity in the germination of Australian alpine species. We found surprisingly low photoperiod sensitivity in the germination of these alpine species (Figure 4e). This might be good news, as photoperiod requirements are less likely to impede the ability of these alpine communities to adapt to new conditions. However, temperature‐related phenology shifts are not always adaptive, and a lack of germination photoperiod sensitivity could leave seedlings at risk of frost damage from exposure to cold snaps after germinating too early under early spring/summer conditions, particularly given the earlier loss of protective snow cover (Venn and Morgan 2007).

Plants are often classified according to their critical day length requirements in a categorical way, that is, as short‐day, long‐day or day‐neutral (Mizoguchi et al. 2007). However, species vary substantially in the stringency of their photoperiod requirements, from a slight tendency to germinate or flower more under longer days to an absolute constraint against germination or flowering under some light regimes. Further, photoperiod sensitivity often varies within species or populations in natural ecosystems (Parker et al. 2021; Wolkovich et al. 2014). In this paper, we developed a novel continuous metric for quantifying the degree of photoperiod sensitivity. Our metric is simple and easy, and the code is openly available at https://anonymous.4open.science/r/Trends‐in‐Germination‐Photoperiod‐326E/. This continuous metric can be used in future photoperiod studies for germination and also for studies quantifying photoperiod sensitivities in a range of phenological events (e.g., leaf‐out, flowering) quantified in experiments or in the field in order to increase our ability to compare across studies in the future.

None of the ideas we had about potential relationships between photoperiod sensitivity and biogeography, phylogeny or environmental conditions were supported by empirical data. We hope that our study might inspire the development and testing of new theories about the mechanisms through which photoperiod sensitivity is favoured. We also hope our study might renew interest in collecting empirical data on photoperiod sensitivity, particularly for species in the southern hemisphere and in understudied habitats and taxonomic groups.

## Author Contributions

**Ashika Perrin:** data curation (lead), formal analysis (lead), methodology (equal), project administration (equal), visualization (equal), writing – original draft (lead), writing – review and editing (equal). **Angela T. Moles:** conceptualization (lead), formal analysis (equal), funding acquisition (lead), investigation (equal), methodology (equal), project administration (equal), resources (equal), supervision (equal), visualization (equal), writing – original draft (equal), writing – review and editing (equal). **Catherine A. Offord:** conceptualization (equal), methodology (equal), project administration (equal), resources (equal), supervision (equal), writing – original draft (equal), writing – review and editing (equal). **Eve Slavich:** data curation (equal), formal analysis (equal), methodology (equal), project administration (equal), visualization (equal), writing – original draft (equal), writing – review and editing (equal). **Karen Zeng:** data curation (equal), formal analysis (equal), methodology (equal), project administration (equal), writing – original draft (equal), writing – review and editing (equal). **Susan E. Everingham:** conceptualization (equal), data curation (equal), formal analysis (equal), investigation (equal), methodology (equal), project administration (equal), supervision (lead), visualization (equal), writing – original draft (equal), writing – review and editing (lead).

## Conflicts of Interest

The authors declare no conflicts of interest.

## Supporting information


**Data S1:** ece371923‐sup‐0001‐Supinfo.docx.

## Data Availability

All data and code are available on Github (https://github.com/SEveringham/Trends‐in‐Germination‐Photoperiod).
