# Supplementary material for: Shining a Light on Patterns of Photoperiod Sensitivity in Germination and Flowering Across Latitudes, Ecosystems and Functional Traits
Source: Ecol Evol. 2025 Aug 27;15(9):e71923. doi: 10.1002/ece3.71923 (PMC12391026; doi:10.1002/ece3.71923)
Supplement: Supplementary file 1 — Data S1: ece371923‐sup‐0001‐Supinfo.docx. [file ECE3-15-e71923-s001.docx]

**Supporting Information**

**Appendix S1**

Information for the collection of seeds used to test germination photoperiod sensitivity in our study and the location of collections

**Table S1.1** Full species list for germination photoperiod quantifications including: date and site of collection, latitude (˚S), longitude (˚E), leaf area index, ecosystem type (alpine or non-alpine), average fresh seed mass (mg), number of Petri dishes and seeds used in each photoperiod treatment (long-day, short-day or equal-day), and the total sample size per species per photoperiod treatment.

| Date of Collection | Site | Latitude (˚S) | Longitude (˚E) | Leaf Area Index (LAI) | Genus species | Family | Growth Form | Alpine or Non-Alpine Habitat | Average Fresh Seed mass (mg) | Number of Petri dishes used in each photoperiod treatment | Seed sample size in each Petri dish | Total Sample size in each photoperiod treatment |
| --- | --- | --- | --- | --- | --- | --- | --- | --- | --- | --- | --- | --- |
|  |  |  |  |  |  |  |  |  |  |  |  |  |
| 25^th^ Feb. 2020 | **Thungutti Swamp** | 30.50 | 152.39 | 4.13 | *Eucalyptus dalrympleana* | Myrtaceae | Tree | Non-Alpine | 0.200 | 1 | 30 | 30 |
|  |  | 30.50 | 152.39 | 4.13 | *Eucalyptus pauciflora* | Myrtaceae | Tree | Non-Alpine | 1.180 | 2 | 15 | 30 |
|  |  | 30.61 | 152.39 | 5.30 | *Leptospermum gregarium* | Myrtaceae | Shrub | Non-Alpine | 0.058 | 1 | 33 | 33 |
|  |  | 30.61 | 152.39 | 5.30 | *Leptospermum polygalifolium* subsp. *cismontanum* | Myrtaceae | Shrub | Non-Alpine | 0.064 | 1 | 33 | 33 |
|  |  | 28.52 | 152.75 | 5.50 | *Tasmannia stipitata* | Winteraceae | Shrub | Non-Alpine | 1.154 | 1 | 33 | 33 |
|  |  |  |  |  |  |  |  |  |  |  |  |  |
| 26^th^ Feb. 2020 | **Dorrigo National Park** | 30.37 | 152.73 | 4.43 | *Acradenia euodiiformis** | Rutaceae | Tree | Non-Alpine | 4.476 | 1 | 6 | 6 |
|  |  | 30.37 | 152.73 | 4.43 | *Acronychia oblongifolia* | Rutaceae | Tree | Non-Alpine | 8.243 | 1 | 13 | 13 |
|  |  | 30.37 | 152.73 | 4.43 | *Cissus hypoglauca+* | Vitaceae | Climber | Non-Alpine | 35.226 | 2 | 20 | 40 |
|  |  | 30.37 | 152.73 | 4.43 | *Cryptocarya microneura** | Lauraceae | Tree | Non-Alpine | 47.520 | 1 | 5 | 5 |
|  |  | 30.37 | 152.73 | 4.43 | *Linospadix monostachyos** | Arecaceae | Shrub | Non-Alpine | 137.740 | 3 | 10 | 30 |
|  |  | 30.37 | 152.73 | 4.43 | *Syzygium crebrinerve** | Myrtaceae | Tree | Non-Alpine | 133.280 | 2 | 11 | 22 |
|  |  |  |  |  |  |  |  |  |  |  |  |  |
| 27^th^ Feb. 2020 | **Rocky Creek in**  **Fortis Creek National Park** | 29.47  29.47 | 152.86  152.86 | 2.45  2.45 | *Banksia aemula ^h^*  *Banksia oblongifolia ^h^* | Proteaceae  Proteaceae | Tree  Tree | Non-Alpine  Non-Alpine | 101.962  27.793 | 1  1 | 4  14 | 4  14 |
|  |  | 29.47 | 152.86 | 2.45 | *Hakea dactyloides* | Proteaceae | Shrub | Non-Alpine | 8.200 | 2 | 7 | 14 |
|  |  | 29.47 | 152.86 | 2.45 | *Petrophile pulchella* | Proteaceae | Shrub | Non-Alpine | 0.259 | 1 | 33 | 33 |
|  |  |  |  |  |  |  |  |  |  |  |  |  |
| 27^th^ Feb. 2020 | **Chambigne Nature Reserve** | 29.81 | 152.78 | 2.67 | *Leptospermum arachnoides* | Myrtaceae | Shrub | Non-Alpine | 0.080 | 1 | 30 | 30 |
|  |  |  |  |  |  |  |  |  |  |  |  |  |
| 28^th^ Feb. 2020 | **Mallanganee National Park** | 29.81 | 152.78 | 2.27 | *Alchornea ilicifolia** | Euphorbiaceae | Shrub | Non-Alpine | 11.248 | 1 | 15 | 15 |
|  |  | 29.81 | 152.78 | 5.00 | *Austrosteenisia blackii ^s^*+ | Fabaceae | Climber | Non-Alpine | 17.584 | 2 | 20 | 40 |
|  |  | 29.81 | 152.78 | 5.00 | *Cissus hypoglauca** | Vitaceae | Climber | Non-Alpine | 97.924 | 3 | 11 | 33 |
|  |  | 29.81 | 152.78 | 5.00 | *Croton insularis* | Euphorbiaceae | Tree | Non-Alpine | 10.638 | 1 | 11 | 11 |
|  |  | 29.81 | 152.78 | 5.00 | *Myrsine howittiana** | Primulaceae | Tree | Non-Alpine | 27.660 | 3 | 12 | 36 |
|  |  |  |  |  |  |  |  |  |  |  |  |  |
| 29^th^ Feb. 2020 | **Border Ranges National Park** | 28.38 | 153.06 | 5.00 | *Cissus hypoglauca** | Vitaceae | Climber | Non-Alpine | 22.840 | 3 | 10 | 30 |
|  |  | 28.38 | 153.06 | 5.00 | *Cupaniopsis flagelliformis* | Sapindaceae | Tree | Non-Alpine | 1116.663 | 1 | 6 | 6 |
|  |  | 28.38 | 153.06 | 5.00 | *Ehretia acuminata** | Boraginaceae | Tree | Non-Alpine | 26.646 | 2 | 20 | 20 |
|  |  | 28.38 | 153.06 | 5.00 | *Homalanthus populifolius** | Euphorbiaceae | Tree | Non-Alpine | 23.84 | 2 | 10 | 20 |
|  |  | 28.38 | 153.06 | 5.00 | *Linospadix monostachyos* | Arecaceae | Shrub | Non-Alpine | 169.612 | 2 | 15 | 30 |
|  |  | 28.38 | 153.06 | 5.00 | *Parsonsia induplicata** | Apocynaceae | Climber | Non-Alpine | 1.890 | 2 | 10 | 20 |
|  |  | 28.38 | 153.06 | 5.00 | *Solanum aviculare** | Solanaceae | Shrub | Non-Alpine | 0.234 | 1 | 30 | 30 |
|  |  | 28.38 | 153.06 | 5.00 | *Syzygium oleosum** | Myrtaceae | Tree | Non-Alpine | 139.620 | 4 | 10 | 40 |
|  |  |  |  |  |  |  |  |  |  |  |  |  |
| 1^st^ March 2020 | **Toonumbar National Park** | 28.52 | 152.7479 | 5.50 | *Breynia oblongifolia** | Phyllanthaceae | Shrub | Non-Alpine | 3.923 | 1 | 9 | 9 |
|  |  | 28.52 | 152.7479 | 5.50 | *Cissus Antarctica** | Vitaceae | Climber | Non-Alpine | 63.74 | 3 | 8 | 24 |
|  |  | 28.52 | 152.7479 | 5.50 | *Cordyline petiolaris** | Asteliaceae | Shrub | Non-Alpine | 7.778 | 2 | 16 | 32 |
|  |  | 28.52 | 152.7479 | 5.50 | *Geitonoplesium cymosum** | Luzuriagaceae | Climber | Non-Alpine | 10.235 | 1 | 11 | 11 |
|  |  | 28.52 | 152.7479 | 5.50 | *Glochidion ferdinandi* | Phyllanthaceae | Tree | Non-Alpine | 18.294 | 1 | 5 | 5 |
|  |  | 28.52 | 152.7479 | 5.50 | *Syzygium australe** | Myrtaceae | Tree | Non-Alpine | 63.789 | 1 | 6 | 6 |
|  |  |  |  |  |  |  |  |  |  |  |  |  |
| 3^rd^ March 2020 | **Bald Rock National Park** | 28.85 | 152.04 | 1.53 | *Coronidium boormanii* | Asteraceae | Herb | Non-Alpine | 0.532 | 2 | 15 | 30 |
|  |  | 28.85 | 152.04 | 1.53 | *Isotoma anethifolia* | Campanulaceae | Herb | Non-Alpine | 0.020 | 2 | 15 | 30 |
|  |  | 28.85 | 152.04 | 1.53 | *Kunzea ericoides* | Myrtaceae | Shrub | Non-Alpine | 0.038 | 1 | 33 | 33 |
|  |  | 28.85 | 152.04 | 1.53 | *Muehlenbeckia M.* sp. *Mt Norman** | Polygonaceae | Shrub | Non-Alpine | 10.374 | 1 | 30 | 60 |
|  |  | 28.85 | 152.04 | 1.53 | *Plectranthus suaveolens* | Lamiaceae | Shrub | Non-Alpine | 0.200 | 1 | 30 | 30 |
|  |  | 28.85 | 152.04 | 1.53 | *Pomax umbellata* | Rubiaceae | Shrub | Non-Alpine | 0.735 | 1 | 16 | 16 |
|  |  | 28.85 | 152.04 | 1.53 | *Trachymene incisa* | Araliaceae | Herb | Non-Alpine | 1.072 | 1 | 30 | 30 |
|  |  |  |  |  |  |  |  |  |  |  |  |  |
| 17^th^ March 2020 | **Flat Rock Creek in Royal National Park** | 34.11  34.11 | 151.07  151.07 | 3.37  3.37 | *Acacia ulicifolia* *^s^*  *Hakea dactyloides* | Fabaceae  Proteaceae | Shrub  Shrub | Non-Alpine  Non-Alpine | 10.886  27.504 | 2  4 | 15  5 | 30  20 |
|  |  | 34.11 | 151.07 | 3.37 | *Hakea sericea* | Proteaceae | Shrub | Non-Alpine | 34.365 | 2 | 5 | 10 |
|  |  | 34.11 | 151.07 | 3.37 | *Leptospermum polygalifolium* | Myrtaceae | Shrub | Non-Alpine | 0.092 | 1 | 36 | 36 |
|  |  |  |  |  |  |  |  |  |  |  |  |  |
| 17^th^ March 2020 | **Coast Walk in Royal National Park** | 34.10 | 151.16 | 1.37 | *Allocasuarina littoralis* | Casuarinaceae | Tree | Non-Alpine | 2.356 | 2 | 15 | 30 |
|  |  | 34.10 | 151.16 | 1.37 | *Banksia serrata ^h^* | Proteaceae | Tree | Non-Alpine | 74.512 | 1 | 10 | 10 |
|  |  | 34.10 | 151.16 | 1.37 | *Epacris microphylla** | Ericaceae | Shrub | Non-Alpine | 0.044 | 1 | 30 | 30 |
|  |  | 34.10 | 151.16 | 1.37 | *Leptospermum juniperinum* | Myrtaceae | Shrub | Non-Alpine | 0.152 | 1 | 33 | 33 |
|  |  | 34.10 | 151.16 | 1.37 | *Leptospermum laevigatum* | Myrtaceae | Shrub | Non-Alpine | 0.304 | 1 | 40 | 40 |
|  |  |  |  |  |  |  |  |  |  |  |  |  |
| 16^th^ March 2020  (delivered from Queensland) | **Rainforest** | 17.46  17.46 | 145.48  145.47 | 5.23  5.33 | *Acronychia crassipetala*  *Alpinia arctiflora* | Rutaceae  Zingiberaceae | Tree  Shrub | Non-Alpine  Non-Alpine | 8.958  11.996 | 1  2 | 8  20 | 8  40 |
|  |  | 17.41 | 145.70 | 5.37 | *Breynia cernua* | Phyllanthaceae | Shrub | Non-Alpine | 13.034 | 3 | 10 | 30 |
|  |  | 17.34 | 145.58 | 4.50 | *Ficus benjamina* | Moraceae | Tree | Non-Alpine | 0.184 | 1 | 30 | 30 |
|  |  | 16.63 | 145.31 | 5.40 | *Ganophyllum falcatum** | Sapindaceae | Tree | Non-Alpine | 215.736 | 4 | 10 | 40 |
|  |  | 16.62 | 145.31 | 5.47 | *Myrsine variabilis** | Primulaceae | Shrub | Non-Alpine | 40.782 | 2 | 20 | 40 |
|  |  | 17.42 | 145.70 | 5.13 | *Neolitsea dealbata* | Lauraceae | Tree | Non-Alpine | 208.06 | 2 | 15 | 30 |
|  |  | 17.45 | 145.48 | 5.27 | *Symplocos wooroonooran/* *Symplocos stawellii* var*. montana* | Symplocaceae | Tree | Non-Alpine | 35.932 | 1 | 28 | 28 |
|  |  | 17.41 | 145.70 | 5.27 | *Triunia erythrocarpa* | Proteaceae | Tree | Non-Alpine | 3085.220 | 2 | 3 | 6 |
|  |  |  |  |  |  |  |  |  |  |  |  |  |
| 17^th^ March 2020 | **Kosciuszko National Park (alpine; >1500m ASL)** | 36.44  36.49 | 148.32  148.29 | 2.13  1.57 | *Acaena ‘Species A’*  *Aciphylla glacialis** | Rosaceae  Apiaceae | Herb  Herb | Alpine  Alpine | 1.486  3.394 | 1  3 | 30  10 | 30  30 |
|  |  | 36.44 | 148.32 | 2.13 | *Carpha nivicola** | Cyperaceae | Herb | Alpine | 1.506 | 1 | 25 | 25 |
|  |  | 36.44 | 148.32 | 2.13 | *Celmisia longifolia* | Asteraceae | Herb | Alpine | 3.198 | 2 | 15 | 30 |
|  |  | 36.44 | 148.32 | 2.13 | *Chionochloa frigida* | Poaceae | Herb | Alpine | 0.981 | 1 | 16 | 16 |
|  |  | 36.44 | 148.32 | 2.13 | *Coronidium scorpiodes* | Asteraceae | Herb | Alpine | 0.136 | 1 | 30 | 30 |
|  |  | 36.47 | 148.27 | 0.90 | *Euphrasia collina* subsp. *diversicolor** | Orobanchaceae | Herb | Alpine | 0.290 | 2 | 20 | 40 |
|  |  | 36.45 | 148.27 | 0.83 | *Leucochrysum albicans* subsp. *alpinum* | Asteraceae | Herb | Alpine | 0.36 | 1 | 9 | 9 |
|  |  | 36.46 | 148.27 | 1.13 | *Oreomyrrhis ciliata/ Chaerophyllum australianum* | Apiaceae | Herb | Alpine | 2.936 | 2 | 15 | 30 |
|  |  | 36.44 | 148.32 | 2.13 | *Plantago muelleri* | Plantaginaceae | Herb | Alpine | 0.394 | 1 | 35 | 35 |
|  |  | 36.44 | 148.32 | 1.80 | *Richea continentis** | Ericaceae | Shrub | Alpine | 0.048 | 1 | 30 | 30 |
|  |  | 36.44 | 148.32 | 2.13 | *Senecio linearifolius* var. *latifolius* | Asteraceae | Herb | Alpine | 0.318 | 1 | 33 | 33 |
|  |  | 36.46 | 148.26 | 0.50 | *Senecio pinnatifolius* subsp. *alpinus* | Asteraceae | Herb | Alpine | 0.272 | 1 | 33 | 33 |
|  |  |  |  |  |  |  |  |  |  |  |  |  |
| 18^th^ March 2020 | **Kosciuszko National Park (non-alpine; <1500m ASL)** | 36.34  36.35 | 148.54  148.52 | 2.47  2.23 | *Acrothamnus hookeri**  *Callistemon sieberi* | Ericaceae  Myrtaceae | Shrub  Shrub | Non-Alpine  Non-Alpine | 8.700  0.036 | 1  1 | 47  47 | 47  47 |
|  |  | 36.44 | 148.52 | 1.90 | *Hakea microcarpa* | Proteaceae | Shrub | Non-Alpine | 5.398 | 3 | 10 | 30 |
|  |  | 36.35 | 148.53 | 2.03 | *Leptospermum grandifolium* | Myrtaceae | Shrub | Non-Alpine | 0.128 | 1 | 21 | 21 |
|  |  | 36.44 | 148.52 | 1.90 | *Leptospermum myrtifolium* | Myrtaceae | Shrub | Non-Alpine | 0.134 | 1 | 30 | 30 |
|  |  | 36.35 | 148.52 | 2.23 | *Ozothamnus hookeri* | Asteraceae | Shrub | Non-Alpine | 0.154 | 1 | 25 | 25 |
|  |  |  |  |  |  |  |  |  |  |  |  |  |
| 20^th^ March 2020 | **Cumberland Plain Woodland** | 34.07 | 150.77 | 1.47 | *Bursaria spinosa* | Pittosporaceae | Shrub | Non-Alpine | 1.152 | 2 | 25 | 50 |
|  |  | 34.07 | 150.77 | 1.47 | *Einadia nutans* | Amaranthaceae | Herb | Non-Alpine | 0.700 | 1 | 30 | 30 |
|  |  | 34.07 | 150.77 | 1.47 | *Eucalyptus ebbaneoensis* subsp. *ebbanoensis* | Myrtaceae | Tree | Non-Alpine | 1.644 | 1 | 40 | 40 |
|  |  | 34.07 | 150.77 | 1.47 | *Eucalyptus websteriana* subsp*. norsemanica* | Myrtaceae | Tree | Non-Alpine | 0.496 | 1 | 35 | 35 |
|  |  | 34.07 | 150.77 | 1.47 | *Plectranthus parviflorus* | Lamiaceae | Shrub | Non-Alpine | 0.262 | 1 | 30 | 30 |
|  |  | 34.07 | 150.77 | 1.47 | *Rhodanthe anthemoides* | Asteraceae | Herb | Non-Alpine | 0.312 | 1 | 30 | 30 |
|  |  |  |  |  |  |  |  |  |  |  |  |  |
| 7^th^ April 2020  (delivered from Queensland) | **Wet sclerophyll Forest** | 17.46  17.46 | 145.47  145.47 | 5.33  5.33 | *Alphitonia petrei**  *Alpinia caerulea* | Rhamnaceae  Zingiberaceae | Tree  Herb | Non-Alpine  Non-Alpine | 7.742  19.724 | 1  2 | 30  15 | 30  30 |
|  |  | 17.43 | 145.40 | 3.03 | *Maytenus disperma** | Celastraceae | Tree | Non-Alpine | 24.954 | 2 | 20 | 40 |
|  |  | 17.39 | 145.39 | 1.83 | *Syncarpia glomulifera* | Myrtaceae | Tree | Non-Alpine | 0.310 | 1 | 15 | 15 |
|  |  | 17.46 | 145.46 | 5.13 | *Syzygium oleosum* | Myrtaceae | Tree | Non-Alpine | 307.930 | 2 | 9 | 18 |
|  |  |  |  |  |  |  |  |  |  |  |  |  |
| 7^th^ April 2020  (delivered from Queensland) | **Woodland** | 17.39  17.39 | 145.38  145.38 | 1.37  1.37 | *Acacia leptoloba ^s^*  *Callitris intratropica** | Fabaceae  Cupressaceae | Shrub  Tree | Non-Alpine  Non-Alpine | 43.222  7.000 | 3  1 | 10  22 | 30  22 |
|  |  | 17.39 | 145.38 | 1.37 | *Chamaecrista concinna* | Fabaceae | Herb | Non-Alpine | 2.306 | 1 | 33 | 33 |
|  |  | 17.38 | 145.26 | 1.30 | *Eucalyptus cloeziana* | Myrtaceae | Tree | Non-Alpine | 1.422 | 1 | 30 | 30 |
|  |  | 17.38 | 145.38 | 1.50 | *Eucalyptus lockyeri* | Myrtaceae | Tree | Non-Alpine | 0.314 | 1 | 41 | 41 |
|  |  | 17.38 | 145.38 | 1.50 | *Eucalyptus shirleyi* | Myrtaceae | Tree | Non-Alpine | 0.795 | 1 | 7 | 7 |
|  |  | 17.37 | 145.31 | 1.63 | *Grevillea glauca** | Proteaceae | Tree | Non-Alpine | 100.188 | 4 | 5 | 20 |
|  |  | 17.38 | 145.25 | 1.53 | *Vachellia bidwillii ^s^* | Fabaceae | Tree | Non-Alpine | 207.676 | 2 | 10 | 20 |
|  |  |  |  |  |  |  |  |  |  |  |  |  |

* Indicates that the species (n=31) was excluded from all of the analyses, as overall germination was insufficient to determine photoperiod sensitivity; germination was <5% in its best treatment, or germinated seeds were <5 across all three photoperiod treatments.

+ Indicates that the species was included in all of the analyses, except for the ‘Growth Form’; due to the small number of total ‘Climbers’ (n=2) they provided insufficient photoperiod information.

*^s^* Indicates that the species was scarified prior to being placed in the germination treatments

*^h^* Indicates that the species was heated in an oven at 120˚C for half an hour to release seeds from pods, prior to being placed in the germination treatment

**Appendix S2**

Information for the data collected to test trends in flowering photoperiod sensitivity

**Table S2.2** Full data list for flowering photoperiod literature search including: latitude (˚N), longitude (˚E), leaf area index, species, family, photoperiodism, 1000 seed weight (g) and the reference from which the record was obtained.

| Latitude (˚N) | Longitude (˚E) | Leaf Area Index (LAI) | Genus species | Family | Photoperiodism | 1000 seed weight (g) | Reference |
| --- | --- | --- | --- | --- | --- | --- | --- |
| 44.31 | -96.78 | 0.540527 | *Panicum virgatum* | Poaceae | Short day | 1.3 | Castro et al 2011 |
| 53.63 | 13.85 | 0.858827 | *Agrostis alba* | Poaceae | Short day | 0.08 | Cooper and Calder 1964 |
| 51.7 | 5.21 | 0.599106 | *Agrostis canina* | Poaceae | Short day | 0.06 | Cooper and Calder 1964 |
| 52.5 | -3.98 | 1.474037 | *Agrostis tenuis* | Poaceae | Day neutral | 0.07 | Cooper and Calder 1964 |
| 51.65 | -4.87 | 1.40012 | *Aira praecox* | Poaceae | Day neutral | 0.16 | Cooper and Calder 1964 |
| 52.21 | 0.12 | 0.583891 | *Alopecurus myosuroides* | Poaceae | Short day | 1.9948 | Cooper and Calder 1964 |
| 51.25 | -0.31 | 1.633784 | *Brachypodium pinnatum* | Poaceae | Day neutral | 3.7 | Cooper and Calder 1964 |
| 51.25 | -0.31 | 1.633784 | *Brachypodium sylvaticum* | Poaceae | Day neutral | 3.56 | Cooper and Calder 1964 |
| 51.25 | -0.31 | 1.633784 | *Briza media* | Poaceae | Day neutral | 0.5 | Cooper and Calder 1964 |
| 51.25 | -0.31 | 1.633784 | *Bromus erectus* | Poaceae | Short day | 4.55 | Cooper and Calder 1964 |
| 51.78 | 11.75 | 0.583891 | *Bromus secalinus* | Poaceae | Day neutral | 8.41 | Cooper and Calder 1964 |
| 51.25 | -0.31 | 1.633784 | *Festuca gigantea* | Poaceae | Day neutral | 1.048 | Cooper and Calder 1964 |
| 49.97 | -5.2 | 1.041579 | *Festuca ovina* | Poaceae | Day neutral | 0.6 | Cooper and Calder 1964 |
| 52.43 | -3.89 | 0.951901 | *Festuca ovina* | Poaceae | Day neutral | 0.6 | Cooper and Calder 1964 |
| 53.39 | -2.6 | 0.276922 | *Holcus lanatus* | Poaceae | Day neutral | 0.3 | Cooper and Calder 1964 |
| -41.6 | 148.04 | 0.630735 | *Phalaris aquatica* | Poaceae | Day neutral | 1.71 | Cooper and Calder 1964 |
| 38.02 | -120.75 | 0.32732 | *Mimulus guttatus* | Phrymaceae | Day neutral | 0.03 | Friedman and Willis 2013 |
| 45.77 | -121.28 | 1.028268 | *Mimulus guttatus* | Phrymaceae | Day neutral | 0.03 | Friedman and Willis 2013 |
| 40.14 | -121.51 | 1.125141 | *Mimulus guttatus* | Phrymaceae | Long day | 0.03 | Friedman and Willis 2013 |
| 38.98 | -122.55 | 0.487676 | *Mimulus guttatus* | Phrymaceae | Long day | 0.03 | Friedman and Willis 2013 |
| 42.65 | -123.53 | 1.787702 | *Mimulus guttatus* | Phrymaceae | Long day | 0.03 | Friedman and Willis 2013 |
| 38.19 | -121.21 | 0.533619 | *Mimulus guttatus* | Phrymaceae | Day neutral | 0.03 | Friedman and Willis 2013 |
| 44.41 | -122.29 | 1.834284 | *Mimulus guttatus* | Phrymaceae | Long day | 0.03 | Friedman and Willis 2013 |
| 38.99 | -123.09 | 0.439992 | *Mimulus guttatus* | Phrymaceae | Day neutral | 0.03 | Friedman and Willis 2013 |
| 38.55 | -120.87 | 0.451457 | *Mimulus guttatus* | Phrymaceae | Day neutral | 0.03 | Friedman and Willis 2013 |
| 38.1 | -120.84 | 0.428818 | *Mimulus guttatus* | Phrymaceae | Day neutral | 0.03 | Friedman and Willis 2013 |
| 37.95 | -120.42 | 0.493989 | *Mimulus guttatus* | Phrymaceae | Long day | 0.03 | Friedman and Willis 2013 |
| 39.27 | -123.24 | 0.745525 | *Mimulus guttatus* | Phrymaceae | Day neutral | 0.03 | Friedman and Willis 2013 |
| 38.04 | -119.99 | 0.858827 | *Mimulus guttatus* | Phrymaceae | Unknown | 0.03 | Friedman and Willis 2013 |
| 42.54 | -124.28 | 1.612905 | *Mimulus guttatus* | Phrymaceae | Long day | 0.03 | Friedman and Willis 2013 |
| 38.92 | -122.58 | 0.513422 | *Mimulus guttatus* | Phrymaceae | Long day | 0.03 | Friedman and Willis 2013 |
| 46.04 | -123.71 | 1.551856 | *Mimulus guttatus* | Phrymaceae | Long day | 0.03 | Friedman and Willis 2013 |
| 45.47 | -121.15 | 0.299138 | *Mimulus guttatus* | Phrymaceae | Long day | 0.03 | Friedman and Willis 2013 |
| 49.03 | -124 | 1.532025 | *Mimulus guttatus* | Phrymaceae | Day neutral | 0.03 | Friedman and Willis 2013 |
| 38.55 | -120.87 | 0.451457 | *Mimulus guttatus* | Phrymaceae | Long day | 0.03 | Friedman and Willis 2013 |
| 20.74 | -121.26 | 2.595869 | *Mimulus guttatus* | Phrymaceae | Long day | 0.03 | Friedman and Willis 2013 |
| 58.07 | -135.89 | 1.041579 | *Mimulus guttatus* | Phrymaceae | Long day | 0.03 | Friedman and Willis 2013 |
| 36.23 | -121.67 | 1.015128 | *Mimulus guttatus* | Phrymaceae | Long day | 0.03 | Friedman and Willis 2013 |
| 41.99 | -118.86 | 0.237316 | *Mimulus guttatus* | Phrymaceae | Long day | 0.03 | Friedman and Willis 2013 |
| 48.63 | -124.46 | 1.154459 | *Mimulus guttatus* | Phrymaceae | Long day | 0.03 | Friedman and Willis 2013 |
| 40.12 | -121.5 | 1.184541 | *Mimulus guttatus* | Phrymaceae | Long day | 0.03 | Friedman and Willis 2013 |
| 43.98 | -124.18 | 2.595869 | *Mimulus guttatus* | Phrymaceae | Long day | 0.03 | Friedman and Willis 2013 |
| 44.41 | -122.16 | 1.698056 | *Mimulus guttatus* | Phrymaceae | Long day | 0.03 | Friedman and Willis 2013 |
| 42.59 | -124.48 | 2.595869 | *Mimulus guttatus* | Phrymaceae | Long day | 0.03 | Friedman and Willis 2013 |
| 45.85 | -124.11 | 2.595869 | *Mimulus guttatus* | Phrymaceae | Long day | 0.03 | Friedman and Willis 2013 |
| 37.06 | -123.75 | 2.595869 | *Mimulus guttatus* | Phrymaceae | Long day | 0.03 | Friedman and Willis 2013 |
| 53.44 | -132.06 | 1.215407 | *Mimulus guttatus* | Phrymaceae | Long day | 0.03 | Friedman and Willis 2013 |
| 40.72 | -121.12 | 0.869944 | *Mimulus guttatus* | Phrymaceae | Long day | 0.03 | Friedman and Willis 2013 |
| 30.88 | 34.79 | 0.19821 | *Spergularia diandra* | Caryophyllaceae | Long day | 0.032 | Gutterman 1997 |
| 68.35 | 18.7 | 0.253079 | *Phippsia algida* | Poaceae | Long day | NA | Heide 1992 |
| 59.67 | 10.83 | 1.882081 | *Carex canescens* | Cyperaceae | Day neutral | 0.357 | Heide 2002 |
| 60.67 | 10.08 | 1.364563 | *Carex canescens* | Cyperaceae | Short day | 0.357 | Heide 2002 |
| -36.5 | 148.5 | 0.64717 | *Carex canescens* | Cyperaceae | Short day | 0.357 | Heide 2002 |
| -36.67 | 148.67 | 0.774853 | *Carex canescens* | Cyperaceae | Short day | 0.357 | Heide 2002 |
| -44.67 | -71.67 | 0.310906 | *Carex canescens* | Cyperaceae | Short day | 0.357 | Heide 2002 |
| 68.83 | 16.5 | 2.595869 | *Carex echinata* | Cyperaceae | Day neutral | 0.81 | Heide 2002 |
| 62.83 | 7.17 | 2.595869 | *Carex echinata* | Cyperaceae | Short day | 0.81 | Heide 2002 |
| 59.42 | 10.67 | 0.599106 | *Carex echinata* | Cyperaceae | Short day | 0.81 | Heide 2002 |
| -36.5 | 148.33 | 1.312914 | *Carex echinata* | Cyperaceae | Short day | 0.81 | Heide 2002 |
| -36.67 | 148.67 | 0.774853 | *Carex echinata* | Cyperaceae | Short day | 0.81 | Heide 2002 |
| -36.58 | 148.83 | 0.57643 | *Carex echinata* | Cyperaceae | Short day | 0.81 | Heide 2002 |
| 68.83 | 16.33 | 1.015128 | *Carex lachenalii* | Cyperaceae | Day neutral | 0.3352 | Heide 2002 |
| 67 | 14.92 | 0.372246 | *Carex lachenalii* | Cyperaceae | Short day | 0.3352 | Heide 2002 |
| 45.17 | 169.83 | 2.595869 | *Carex lachenalii* | Cyperaceae | Short day | 0.3352 | Heide 2002 |
| 60.08 | 12.08 | 1.474037 | *Carex magellanica* | Cyperaceae | Short day | 1.5 | Heide 2002 |
| -63.08 | -71.17 | NA | *Carex magellanica* | Cyperaceae | Short day | 1.5 | Heide 2002 |
| 45.08 | 6.42 | 0.475291 | *Oxyria digyna* | Polygonaceae | Short long day | 0.78 | Heide 2005 |
| 60.58 | 7.5 | 0.115485 | *Oxyria digyna* | Polygonaceae | Short long day | 0.78 | Heide 2005 |
| 68.83 | 16.33 | 1.015128 | *Oxyria digyna* | Polygonaceae | Short long day | 0.78 | Heide 2005 |
| 78.17 | 15.5 | NA | *Oxyria digyna* | Polygonaceae | Short long day | 0.78 | Heide 2005 |
| 62.3 | 9.6 | 0.599106 | *Koenigia islandica* | Polygonaceae | Long day | NA | Heide and Gauslaa 1999 |
| 47.15 | 11.24 | 0.417928 | *Androsace alpina* | Primulaceae | Long day | NA | Keller and Körner 2003 |
| 47.15 | 11.24 | 0.417928 | *Carex curvula* | Cyperaceae | Day neutral | 2 | Keller and Körner 2003 |
| 47.15 | 11.24 | 0.417928 | *Cerastium uniflorum* | Caryophyllaceae | Day neutral | 0.2556 | Keller and Körner 2003 |
| 47.15 | 11.24 | 0.417928 | *Elyna myosuroides* | Cyperaceae | Day neutral | NA | Keller and Körner 2003 |
| 47.15 | 11.24 | 0.417928 | *Erigeron uniflorus* | Asteraceae | Yes | 0.16 | Keller and Körner 2003 |
| 47.15 | 11.24 | 0.417928 | *Festuca af. intercedens* | Poaceae | Long day | NA | Keller and Körner 2003 |
| 47.15 | 11.24 | 0.417928 | *Gentiana bavarica* | Gentianaceae | Day neutral | NA | Keller and Körner 2003 |
| 47.15 | 11.24 | 0.417928 | *Geum reptans* | Rosaceae | Day neutral | 1.083333 | Keller and Körner 2003 |
| 47.15 | 11.24 | 0.417928 | *Gnaphalium supinum* | Asteraceae | Long day | 0.0928 | Keller and Körner 2003 |
| 47.15 | 11.24 | 0.417928 | *Leucanthemopsis alpina* | Asteraceae | Long day | 0.3801 | Keller and Körner 2003 |
| 47.15 | 11.24 | 0.417928 | *Linaria alpina* | Plantaginaceae | Long day | 0.329 | Keller and Körner 2003 |
| 47.15 | 11.24 | 0.417928 | *Luzula spicata* | Juncaceae | Long day | 0.27 | Keller and Körner 2003 |
| 47.15 | 11.24 | 0.417928 | *Minuartia sedoides* | Caryophyllaceae | Day neutral | NA | Keller and Körner 2003 |
| 47.15 | 11.24 | 0.417928 | *Oxyria digyna* | Polygonaceae | Long day | 0.78 | Keller and Körner 2003 |
| 47.15 | 11.24 | 0.417928 | *Poa laxa* | Poaceae | Long day | 0.3588 | Keller and Körner 2003 |
| 47.15 | 11.24 | 0.417928 | *Potentilla frigida* | Rosaceae | Yes | NA | Keller and Körner 2003 |
| 47.15 | 11.24 | 0.417928 | *Ranunculus glacialis* | Ranunculaceae | Day neutral | 0.5078 | Keller and Körner 2003 |
| 47.15 | 11.24 | 0.417928 | *Saxifraga bryoides* | Saxifragaceae | Long day | 0.0442 | Keller and Körner 2003 |
| 47.15 | 11.24 | 0.417928 | *Saxifraga oppositifolia* | Saxifragaceae | Day neutral | 0.11 | Keller and Körner 2003 |
| 47.15 | 11.24 | 0.417928 | *Saxifraga seguieri* | Saxifragaceae | Day neutral | 0.04 | Keller and Körner 2003 |
| 47.15 | 11.24 | 0.417928 | *Sedum alpestre* | Crassulaceae | Long day | 0.041 | Keller and Körner 2003 |
| 47.15 | 11.24 | 0.417928 | *Sibbaldia procumbens* | Rosaceae | Yes | 0.4 | Keller and Körner 2003 |
| 44.14 | -122.58 | 1.676356 | *Mimulus alsinoides* | Phrymaceae | Long day | NA | Kooyers et al 2017 |
| -28.16 | 148.23 | 0.381945 | *Pycnosorus thompsonianus* | Asteraceae | Long day | NA | Krisantini et al 2015 |
| -28.16 | 148.23 | 0.381945 | *Rhodanthe floribunda* | Asteraceae | Long day | 0.39 | Krisantini et al 2015 |
| 33.88 | -81 | 1.698056 | *Microstegium vimineum* | Poaceae | Short day | NA | Novy et al 2013 |
| 35.95 | -79.16 | 1.720037 | *Microstegium vimineum* | Poaceae | Short day | NA | Novy et al 2013 |
| 38.76 | -77.18 | 0.755175 | *Microstegium vimineum* | Poaceae | Short day | NA | Novy et al 2013 |
| 38.9 | -76.39 | 2.595869 | *Microstegium vimineum* | Poaceae | Short day | NA | Novy et al 2013 |
| 39.07 | -77.39 | 1.32991 | *Microstegium vimineum* | Poaceae | Short day | NA | Novy et al 2013 |
| 39.63 | -75.71 | 1.382227 | *Microstegium vimineum* | Poaceae | Short day | NA | Novy et al 2013 |
| 39.77 | -79.98 | 1.834284 | *Microstegium vimineum* | Poaceae | Short day | NA | Novy et al 2013 |
| 40.45 | -79.82 | 1.296136 | *Microstegium vimineum* | Poaceae | Short day | NA | Novy et al 2013 |
| 40.62 | -74.89 | 1.612905 | *Microstegium vimineum* | Poaceae | Short day | NA | Novy et al 2013 |
| 41.35 | -73.13 | 1.764856 | *Microstegium vimineum* | Poaceae | Short day | NA | Novy et al 2013 |
| 51.69 | 14.32 | 1.382227 | *Ambrosia artemisiifolia* | Asteraceae | Short day | 3.63 | Scalone et al 2016 |
| 47.44 | 18.92 | 0.726592 | *Ambrosia artemisiifolia* | Asteraceae | Short day | 3.63 | Scalone et al 2016 |
| 46.4 | 17.9 | 1.215407 | *Ambrosia artemisiifolia* | Asteraceae | Short day | 3.63 | Scalone et al 2016 |
| 47.23 | 5.25 | 0.939736 | *Ambrosia artemisiifolia* | Asteraceae | Short day | 3.63 | Scalone et al 2016 |
| 45.37 | 9.03 | 0.690156 | *Ambrosia artemisiifolia* | Asteraceae | Short day | 3.63 | Scalone et al 2016 |
| 45.17 | 73.81 | 0.211376 | *Ambrosia artemisiifolia* | Asteraceae | Short day | 3.63 | Scalone et al 2016 |
| 44.91 | 0.68 | 1.26322 | *Ambrosia artemisiifolia* | Asteraceae | Short day | 3.63 | Scalone et al 2016 |
| 44.75 | 17.93 | 1.858029 | *Ambrosia artemisiifolia* | Asteraceae | Short day | 3.63 | Scalone et al 2016 |
| 43.94 | 4.36 | 0.976705 | *Ambrosia artemisiifolia* | Asteraceae | Short day | 3.63 | Scalone et al 2016 |
| 38.02 | 84.58 | 0.157247 | *Ambrosia artemisiifolia* | Asteraceae | Short day | 3.63 | Scalone et al 2016 |
| 42.45 | -76.47 | 1.125141 | *Plantago lanceolata* | Plantaginaceae | Long day | 1.3 | Snyder 1948 |
| 38.51 | 84.09 | 0.147453 | *Solidago shortii* | Asteraceae | Short day | NA | Walck et al 1999 |
| 45.56 | -84.68 | 1.474037 | *Silene latifolia* | Caryophyllaceae | Short day | 0.81 | Wang 2007 |
| -25.03 | 31.93 | 0.417928 | *Hibiscus engleri* | Malvaceae | Short day | 3.8948 | Warner and Erwin 2001 |
| 51.96 | 4.13 | 0.276922 | *Persicaria lapathifolia* | Polygonaceae | Short day | 1.49 | Wisskirchen 2006 |
| 51.03 | 7.63 | 1.296136 | *Alopecurus aequalis* | Poaceae | Long day | 0.168 | Wisskirchen 2006 |
| 50.66 | 6.81 | 0.599106 | *Atriplex prostrata* | Amaranthaceae | Short day | 4 | Wisskirchen 2006 |
| 50.83 | 6.96 | 0.69909 | *Bidens cernua* | Asteraceae | Short day | 3.33 | Wisskirchen 2006 |
| 50.69 | 6.94 | 1.028268 | *Bidens tripartita* | Asteraceae | Short day | 2.3 | Wisskirchen 2006 |
| 50.82 | 6.99 | 0.540527 | *Chenopodium ficifolium* | Amaranthaceae | Day neutral | 0.369 | Wisskirchen 2006 |
| 50.76 | 7.09 | 1.015128 | *Chenopodium glaucum* | Amaranthaceae | Short day | 0.232 | Wisskirchen 2006 |
| 51.96 | 4.13 | 0.276922 | *Persicaria lapathifolia ssp. lapathifolia* | Polygonaceae | Short day | NA | Wisskirchen 2006 |
| 51.96 | 4.13 | 0.276922 | *Persicaria lapthifolia ssp. brittingeri* | Polygonaceae | Short day | NA | Wisskirchen 2006 |
| 50.01 | 7.12 | 1.002155 | *Plantago major* | Plantaginaceae | Long day | 0.143 | Wisskirchen 2006 |
| 50.87 | 6.4 | 0.881205 | *Ranunculus sceleratus* | Ranunculaceae | Long day | 0.2 | Wisskirchen 2006 |
| 51.03 | 7.63 | 1.296136 | *Rorippa paulstris* | Brassicaceae | Long day | NA | Wisskirchen 2006 |
| 51.09 | 6.84 | 0.64717 | *Rumex palustris* | Polygonaceae | Long day | NA | Wisskirchen 2006 |
| 42.02 | -93.58 | 0.475291 | *Amaranthus tuberculatus* | Amaranthaceae | Short day | 0.229 | Wu and Owen 2014 |
| -22.92 | -47.05 | 0.263035 | *Hyptis brevipes* | Lamiaceae | Short day | NA | Zaidan et al 1991 |

**Reference list for data in Table S2.2**

Castro JC, Boe A, Lee DK. 2011. A Simple System for Promoting Flowering of Upland Switchgrass in the Greenhouse. *Crop Science* 51: 2607–2614.

Cooper JP, Calder DM. 1964. The Inductive Requirements for Flowering of Some Temperate Grasses. *Grass and Forage Science* 19: 6–14.

Friedman J, Willis JH. 2013. Major QTLs for critical photoperiod and vernalization underlie extensive variation in flowering in the Mimulus guttatus species complex. *New Phytologist* 199: 571–583.

Gutterman Y. 1997. Effect of daylength on flowering and seed morphology of Spergularia diandraoccurring in the Negev Desert, Israel. *Journal of Arid Environments* 36: 611–622.

Heide OM. 1992. Flowering strategies of the high-arctic and high-alpine snow bed grass species Phippsia algida. *Physiologia Plantarum* 85: 606–610.

Heide OM. 2002. Climatic flowering requirements of bipolar sedges, Carex spp., and the feasibility of their trans-equatorial migration by mountain-hopping. *Oikos* 99: 352–362.

Heide OM. 2005. Ecotypic Variation among European Arctic and Alpine Populations of Oxyria digyna. *Arctic, Antarctic, and Alpine Research* 37: 233–238.

Keller F, Korner C. 2003. The Role of Photoperiodism in Alpine Plant Development. *Arctic, Antarctic, and Alpine Research* 35: 361–368.

Kooyers NJ, James B, Blackman BK. 2017. Competition drives trait evolution and character displacement between Mimulus species along an environmental gradient. *Evolution* 71: 1205–1221.

Krisantini, Wickramasinghe P, Wickramasinghe V, Johnston M. 2015. Effect of photoperiod and temperature on flowering of Rhodanthe floribunda and Pycnosorus thompsonianus. *New Zealand Journal of Crop and Horticultural Science* 43: 275–281.

Novy A, Flory S l., Hartman JM. 2013. Evidence for rapid evolution of phenology in an invasive grass. *Journal of Evolutionary Biology* 26: 443–450.

Scalone R, Lemke A, Štefanić E, Kolseth A-K, Rašić S, Andersson L. 2016. Phenological Variation in Ambrosia artemisiifolia L. Facilitates Near Future Establishment at Northern Latitudes. *PLOS ONE* 11: e0166510.

Snyder WE. 1948. Mechanism of the Photoperiodic Response of Plantago lanceolata L., A Long-Day Plant. *American Journal of Botany* 35: 520–525.

Walck JL, Baskin JM, Baskin CC. 1999. Ecology of the Endangered Species Solidago shortii. VII. Survivorship and Flowering, and Comparison with Common, Geographically-Widespread Solidago Species. *The Journal of the Torrey Botanical Society* 126: 124–132.

Wang X. 2007. Gender-specific flowering responses to day length in the dioecious plant Silene latifolia at different temperatures. *Sexual Plant Reproduction* 20: 45–50.

Warner RM, Erwin JE. 2001. Variation in floral induction requirements of Hibiscus sp. *Journal of the American Society for Horticultural Science* 126: 262–268.

Wisskirchen R. 2006. An experimental study on the growth and flowering of riparian pioneer plants under long- and short-day conditions. *Flora - Morphology, Distribution, Functional Ecology of Plants* 201: 3–23.

Wu C, Owen MDK. 2014. When Is the Best Time to Emerge: Reproductive Phenology and Success of Natural Common Waterhemp (Amaranthus rudis) Cohorts in the Midwest United States? *Weed Science* 62: 107–117.

Zaidan LBP, Dietrich SMC, Schwabe WW. 1991. Effects of temperature and photoperiod on flowering in Hyptis brevipes. *Physiologia Plantarum* 81: 221–226.

**APPENDIX S3**

Results for each species’ germination test to determine the photoperiod sensitivity metric of germation.

**Table S3.1** Species’ seed germination data per photoperiod treatment and *p*-values of pairwise comparisons of between-group relationships. The applied photoperiod treatments include: 12 hours of light, 12 hour of darkness (12L/12D); 18 hours of light, 6 hours of darkness (18L/6D); and 6 hours of light, 18 hours of darkness (6L/18D).

| Genus species | Total Germination per Treatment | | | Photoperiod Sensitivity Metric: |  | *p*-Values |  |  |
| --- | --- | --- | --- | --- | --- | --- | --- | --- |
|  | **12L/12D (ID):** | **18L/6D (LD):** | **6L/18D**  **(SD):** |  | **ID-LD** | **ID-SD** | **LD-SD** |  |
|  |  |  |  |  |  |  |  |  |
| *Acacia leptoloba* | 30/30 | 30/30 | 30/30 | 0.00 | 1.00 | 1.00 | 1.00 |  |
| *Acacia ulicifolia* | 30/30 | 29/30 | 30/30 | 1.38 | 0.66 | l0.97 | 0.66 |  |
| *Acaena ‘Species A’/ Acaena novae-zelandiae* | 13/30 | 22/30 | 22/30 | 0.94 | 0.07 | 0.07 | 1.00 |  |
| *Acronychia crassipetala* | 2/8 | 1/8 | 0/8 | 1.33 | 0.92 | 0.47 | 0.70 |  |
| *Acronychia oblongifolia* | 6/13 | 0/13 | 4/13 | 2.52 | 0.09 | 0.81 | 0.18 |  |
| *Allocasuarina littoralis* | 14/30 | 16/30 | 20/30 | 0.55 | 0.90 | 0.29 | 0.56 |  |
| *Alpinia arctiflora* | 5/40 | 2/40 | 8/40 | 1.01 | 0.49 | 0.61 | 0.15 |  |
| *Alpinia caerulea* | 3/30 | 2/30 | 0/30 | 1.63 | 0.96 | 0.61 | 0.15 |  |
| *Austrosteenisia blackii* | 40/40 | 40/40 | 38/40 | 1.92 | 0.95 | 0.38 | 0.40 |  |
| *Banksia aemula* | 3/4 | 4/4 | 4/4 | 0.88 | 0.80 | 0.80 | 1.00 |  |
| *Banksia oblongifolia* | 14/14 | 13/14 | 14/14 | 1.41 | 0.65 | 0.97 | 0.65 |  |
| *Banksia serrata* | 10/10 | 10/10 | 8/10 | 2.04 | 0.95 | 0.36 | 0.38 |  |
| *Breynia cernua* | 0/30 | 0/30 | 7/30 | 3.18 | 0.95 | 0.06+ | 0.09 |  |
| *Bursaria spinosa* | 24/50 | 18/50 | 11/50 | 0.81 | 0.62 | 0.02* | 0.20 |  |
| *Callistemon sieberi* | 23/33 | 16/33 | 25/33 | 0.85 | 0.20 | 0.82 | 0.07+ |  |
| *Celmisia longifolia* | 24/30 | 25/30 | 25/30 | 0.15 | 0.95 | 0.95 | 1.00 |  |
| *Chamaecrista concinna* | 4/33 | 18/33 | 14/33 | 1.42 | 0.002* | 0.03* | 0.59 |  |
| *Chionochloa frigida* | 7/16 | 4/16 | 4/16 | 0.56 | 0.58 | 0.59 | 1.00 |  |
| *Cissus hypoglauca* | 3/40 | 4/40 | 10/40 | 0.95 | 0.97 | 0.11 | 0.20 |  |
| *Coronidium boormanii* | 21/30 | 21/30 | 22/30 | 0.12 | 0.96 | 0.96 | 1.00 |  |
| *Coronidium scorpiodes* | 19/30 | 22/30 | 16/30 | 0.59 | 0.68 | 0.71 | 0.27 |  |
| *Croton insularis* | 5/11 | 6/11 | 6/11 | 0.26 | 0.92 | 1.00 | 0.91 |  |
| *Cupaniopsis flagelliformis* | 6/6 | 6/6 | 4/6 | 2.15 | 0.95 | 0.33 | 0.35 |  |
| *Einadia nutans* | 1/30 | 8/30 | 1/30 | 1.73 | 0.05+ | 0.98 | 0.05+ |  |
| *Eucalyptus cloeziana* | 4/30 | 4/30 | 2/30 | 0.53 | 1.00 | 0.71 | 0.71 |  |
| *Eucalyptus dalrympleana* | 29/30 | 30/30 | 29/30 | 1.05 | 0.73 | 0.99 | 0.72 |  |
| *Eucalyptus ebbaneoensis* subsp. *ebbanoensis* | 38/40 | 38/40 | 40/40 | 1.47 | 0.99 | 0.48 | 0.47 |  |
| *Eucalyptus lockyeri* | 32/41 | 32/41 | 35/41 | 0.38 | 1.00 | 0.69 | 0.68 |  |
| *Eucalyptus pauciflora* | 10/30 | 2/30 | 3/30 | 1.24 | 0.06+ | 0.12 | 0.90 |  |
| *Eucalyptus shirleyi* | 5/7 | 4/7 | 5/7 | 0.42 | 0.87 | 1.00 | 0.86 |  |
| *Eucalyptus websteriana* subsp*. norsemanica* | 33/35 | 34/35 | 34/35 | 0.38 | 0.89 | 0.89 | 1.00 |  |
| *Ficus benjamina* | 23/30 | 22/30 | 20/30 | 0.32 | 0.97 | 0.69 | 0.85 |  |
| *Glochidion ferdinandii* | 1/5 | 1/5 | 2/5 | 0.61 | 0.99 | 0.82 | 0.81 |  |
| *Hakea dactyloides* (R.N.P) | 19/20 | 19/20 | 16/20 | 1.09 | 0.99 | 0.37 | 0.37 |  |
| *Hakea dactyloides*  (R.C) | 10/14 | 6/14 | 5/14 | 0.91 | 0.38 | 0.21 | 0.92 |  |
| *Hakea microcarpa* | 2/10 | 0/10 | 2/10 | 0.46 | 0.64 | 0.64 | 1.00 |  |
| *Hakea sericea* | 8/10 | 9/10 | 7/10 | 0.79 | 0.82 | 0.85 | 0.57 |  |
| *Isotoma anethifolia* | 2/30 | 7/30 | 9/30 | 1.17 | 0.10 | 0.10 | 1.00 |  |
| *Kunzea ericoides* | 4/33 | 16/33 | 11/33 | 1.22 | 0.009* | 0.16 | 0.42 |  |
| *Leptospermum arachnoides* | 9/30 | 5/30 | 9/30 | 0.57 | 0.48 | 1.00 | 0.47 |  |
| *Leptospermum grandifolium* | 5/21 | 7/21 | 12/21 | 0.94 | 0.85 | 0.10 | 0.28 |  |
| *Leptospermum gregarium* | 26/33 | 19/33 | 19/33 | 0.73 | 0.20 | 0.20 | 1.00 |  |
| *Leptospermum juniperinum* | 4/33 | 5/33 | 9/33 | 0.66 | 0.96 | 0.31 | 0.47 |  |
| *Leptospermum laevigatum* | 11/40 | 12/40 | 8/40 | 0.38 | 0.96 | 0.72 | 0.57 |  |
| *Leptospermum myrtifolium* | 15/30 | 24/30 | 30/30 | 3.24 | 0.06+ | 0.02* | 0.13 |  |
| *Leptospermum polygalifolium* | 24/36 | 19/36 | 24/36 | 0.45 | 0.47 | 1.00 | 0.47 |  |
| *Leptospermum polygalifolium* subsp. *cismontanum* | 25/33 | 30/33 | 30/33 | 0.80 | 0.30 | 0.30 | 1.00 |  |
| *Leucochrysum albicans* subsp. *alpinum* | 1/9 | 1/9 | 1/9 | 0.00 | 1.00 | 1.00 | 1.00 |  |
| *Linospadix monostachyos* | 9/30 | 2/30 | 3/30 | 1.12 | 0.10 | 0.20 | 0.90 |  |
| *Neolitsea dealbata* | 5/30 | 5/30 | 9/30 | 0.58 | 1.00 | 0.47 | 0.46 |  |
| *Oreomyrrhis ciliata/ Chaerophyllum australianum* | 9/30 | 7/30 | 13/30 | 0.63 | 0.82 | 0.54 | 0.25 |  |
| *Ozothamnus hookeri* | 8/25 | 14/25 | 8/25 | 0.76 | 0.23 | 1.00 | 0.22 |  |
| *Petrophile pulchella* | 20/33 | 16/33 | 13/33 | 0.56 | 0.64 | 0.23 | 0.74 |  |
| *Plantago muelleri* | 14/35 | 25/35 | 24/35 | 0.94 | 0.03* | 0.06+ | 0.96 |  |
| *Plectranthus parviflorus* | 11/30 | 10/30 | 4/30 | 0.94 | 0.98 | 0.12 | 0.19 |  |
| *Plectranthus suaveolens* | 27/30 | 25/30 | 28/30 | 0.66 | 0.73 | 0.88 | 0.49 |  |
| *Pomax umbellata* | 15/16 | 10/16 | 13/16 | 1.17 | 0.14 | 0.72 | 0.47 |  |
| *Rhodanthe anthemoides* | 30/30 | 28/30 | 30/30 | 1.94 | 0.38 | 0.95 | 0.40 |  |
| *Senecio linearifolius* var. *latifolius* | 29/33 | 31/33 | 32/33 | 0.78 | 0.77 | 0.46 | 0.86 |  |
| *Senecio pinnatifolius* subsp. *alpinus* | 26/33 | 25/33 | 27/33 | 0.24 | 0.95 | 0.95 | 0.83 |  |
| *Syncarpia glomulifera* | 2/15 | 3/15 | 5/15 | 0.68 | 0.94 | 0.47 | 0.70 |  |
| *Symplocos wooroonooran/* *Symplocos stawellii* var*. montana* | 8/28 | 5/28 | 5/28 | 0.38 | 0.85 | 0.66 | 0.94 |  |
| *Syzygium oleosum* | 18/18 | 17/18 | 18/18 | 1.40 | 0.66 | 0.97 | 0.65 |  |
| *Tasmannia stipitata* | 6/33 | 8/33 | 3/33 | 0.77 | 0.80 | 0.55 | 0.26 |  |
| *Trachymene incisa* | 14/30 | 19/30 | 17/30 | 0.44 | 0.44 | 0.76 | 0.86 |  |
| *Triunia erythrocarpa* | 3/6 | 1/6 | 2/6 | 0.78 | 0.57 | 0.92 | 0.82 |  |
| *Vachellia bidwillii* | 20/20 | 20/20 | 20/20 | 0.00 | 1.00 | 1.00 | 1.00 |  |

* Indicates species with statistical significance between the respective photoperiod treatments where *p* < 0.05.

**Appendix S4**

Supplementary Analysis using a One-Step model approach to test environmental and ecological trends in photoperiod sensitivity

Methods

We performed a supplementary analysis using a Bayesian binomial generalised linear mixed model with data from all species (except those we excluded due to low germination across treatments), with a treatment (photoperiod exposure/daylength) by each environmental and ecological variable (*X*) interaction. The slope of the photo period sensitivity metric is a non-linear function of the interaction coefficients, rather than a contrast from a model summary. We ran a sensitivity analysis, calculating the Slope and CI of the Slope of PPS, for each environmental and ecological variable using this “interaction model” approach, and compared it to the two-stage analysis approach reported in the main text where the photo period sensitivity metrics was calculated separately per species, and then these photoperiod sensitivity metrics were regressed against the particular environmental and ecological variables.

In this approach, for each ecological and environmental variable (*X*), the germination rate (success/ failure) is the response variable, photoperiod exposure or daylength (with three treatments: 12hr light/12hr dark, 8hr light/16hr dark or 16hr light/8hr dark) is a factor predictor which interacts with *X,* and there is a random intercept and slope for photo period exposure per species (Equation S4.1).

*logit(p_ij) = β₀ + β₁X₁ᵢ + β₂Z₂ᵢⱼ + β₃Z₃ᵢⱼ + β₄(X₁ᵢ × Z₂ᵢⱼ) + β₅(X₁ᵢⱼ × Z₃ᵢⱼ) + u₀ᵢ + u₁ᵢZ₂ᵢⱼ + u₂ᵢX₃ᵢⱼ*

**Equation S4.1** predicted germination  probability as a function of the regression coefficients Where:  *p_ij* is the probability of success for observation *j* of species *i*, *X₁ᵢ* is the *X* variable (ecological or environmental variable), *Z₂ᵢⱼ* is the dummy variable for the second level of the photo period exposure, *Z₃ᵢⱼ* is the dummy variable for the third level of the photo period exposure, *β₀* is the intercept (corresponding to the baseline level of the treatment), *β₁* is the coefficient for the *X* variable, *β₂* is the coefficient for the second level of the photo period exposure, *β₃* is the coefficient for the third level of the photo period exposure, *β₄* is the coefficient for the interaction between *X* and the second level of photo period exposure, *β₅* is the coefficient for the interaction between *X* and the third level of photo period exposure, *u₀ᵢ* is the random intercept for species *i*, *u₁ᵢ* is the random slope for the second level of photo period exposure for species *i, u₂ᵢ* is the random slope for the third level of photo period exposure for species *i*.

We then calculated the slope of photoperiod sensitivity metric. The photoperiod sensitivity metric is defined as the sum of the squared differences between the logit-germination rates in each photo-period exposure. The slope changes in germination rate as *X* (environmental and ecological variable) changes are influenced by *β₁, β₄, β₅,* but only *β₄, β₅* are associated with differences in slope changes due to photo period exposure. *β₄* represents the difference between exposure 2, and 1 (in their slope with *X*); *β₅* represents the difference between exposure 3, and 1 (in their slope with *X*); whilst $\beta_{4}-\beta_{5}$ represents the difference between exposure 2 and 3 (in their slope with *X*) (Equation S4.2).

*Slope (photoperiod sensitivity versus* X) *=* $\sqrt{{( \beta}_{4}^{2}+\beta_{5}^{2}+ {(\beta_{4}-\beta_{5})}^{2})/3}$

**Equation S4.2** Slope of the photoperiod sensitivity metric against each environmental/ecological variable.

After checking model assumptions, we assessed uncertainty in the slope by simulating new coefficients from the multivariate normal distribution estimated for the coefficients using simulate_parameters() from the ‘parameters’ package in R (Lüdecke et al., 2020). Over 1000 iterations, we recalculated the Slope (photoperiod sensitivity versus *X*) and estimated the standard error by taking the standard deviations from this slope. From here, we were able to estimate 95% confidence intervals using Slope +/- 1.96*SE(Slope).

Results

We found results that mirrored our main analyses showing no strong correlations between germination photoperiod sensitivity and any of the measured ecological or environmental variables (Tables S4.1, S4.2). However, leaf area index showed a stronger positive correlation with photoperiod sensitivity in the Bayesian one-step framework than in the frequentist two-step framework.

**Table S4.1** Comparison of results from the Bayesian binomial generalised linear mixed models (one-step analysis) and the generalised linear mixed models (two-step analysis) to test relationships between each continuous environmental/ecological predictor variable and germination photoperiod sensitivity

| Environmental/Ecological variable | Slope | | Standard Error | | | Confidence intervals | |
| --- | --- | --- | --- | --- | --- | --- | --- |
|  | **Bayesian GLM** | **GLM** | **Bayesian GLM** | **GLM** | | **Bayesian GLM** | **GLM** |
| Latitude | 0.074 | 0.001 | 0.067 | | 0.007 | [-0.057, 0.205] | [-0.013, 0.015] |
| Seed Mass | 0.091 | 0.0001 | 0.058 | | 0.0003 | [-0.023, 0.205] | [-0.0005, 0.0007] |
| Leaf Area Index | 0.239 | 0.046 | 0.099 | | 0.036 | [0.045, 0.433] | [-0.024, 0.118] |

**Table S4.2** Results from the Bayesian binomial generalised linear mixed models to test relationships between each factor environmental/ecological predictor variable and germination photoperiod sensitivity

| Environmental/Ecological variable | Slope or contrasts between levels (Bayesian) | | Standard Error | | Confidence Intervals | |
| --- | --- | --- | --- | --- | --- | --- |
|  | **Bayes GLM** | **GLM** | **Bayes GLM** | **GLM** | **Bayes GLM** | **GLM** |
| Alpine vs Non-Alpine Habitats | 0.409 | 0.158 | 0.234 | 0.104 | [-0.050, 0.868] | [-0.046, 0.362] |
| Growth Form | 0.482 *(Herb – Shrub)*  0.616 *(Herb – Tree)* | 0.169 *(Herb – Shrub)*  -0.018 *(Herb – Tree)* | 0.224 *(Herb – Shrub)*  0.303*(Herb – Tree)* | 0.121 *(Herb – Shrub)*  0.120 *(Herb –Tree)* | [0.043, 0.921] *(Herb – Shrub)*  [0.022, 1.210] *(Herb – Tree)* | [-0.068, 0.406] *(Herb – Shrub)*  [-0.253, 0.217] *(Herb – Tree)* |

References

Lüdecke, D., Ben-Shachar, M. S., Patil, I., & Makowski, D. (2020). Extracting, Computing and Exploring the Parameters of Statistical Models using R. *Journal of Open Source Software*, 5(53), 2445.
